# Genomic and transcriptomic analyses reveal polygenic architecture for ecologically important traits in aspen (*Populus tremuloides* Michx.)

**DOI:** 10.1002/ece3.10541

**Published:** 2023-09-28

**Authors:** Jennifer F. L. Riehl, Christopher T. Cole, Clay J. Morrow, Hilary L. Barker, Carolina Bernhardsson, Kennedy Rubert‐Nason, Pär K. Ingvarsson, Richard L. Lindroth

**Affiliations:** ^1^ Department of Entomology University of Wisconsin‐Madison Madison Wisconsin USA; ^2^ Department of Forest and Wildlife Ecology University of Wisconsin‐Madison Madison Wisconsin USA; ^3^ Department of Ecology and Environmental Science Umeå University Umeå Sweden; ^4^ Department of Plant Biology Swedish University of Agricultural Sciences, Uppsala BioCenter Uppsala Sweden; ^5^ Present address: Office of Student Success Wisconsin Technical College System Madison Wisconsin USA; ^6^ Present address: Department of Organismal Biology, Center for Evolutionary Biology Uppsala University Uppsala Sweden; ^7^ Present address: Division of Natural Sciences University of Maine at Fort Kent Fort Kent Maine USA

**Keywords:** community genetics, differential expression, multilocus association model, omnigenic model, polygenic architecture, salicinoids

## Abstract

Intraspecific genetic variation in foundation species such as aspen (*Populus tremuloides* Michx.) shapes their impact on forest structure and function. Identifying genes underlying ecologically important traits is key to understanding that impact. Previous studies, using single‐locus genome‐wide association (GWA) analyses to identify candidate genes, have identified fewer genes than anticipated for highly heritable quantitative traits. Mounting evidence suggests that polygenic control of quantitative traits is largely responsible for this “missing heritability” phenomenon. Our research characterized the genetic architecture of 30 ecologically important traits using a common garden of aspen through genomic and transcriptomic analyses. A multilocus association model revealed that most traits displayed a highly polygenic architecture, with most variation explained by loci with small effects (likely below the detection levels of single‐locus GWA methods). Consistent with a polygenic architecture, our single‐locus GWA analyses found only 38 significant SNPs in 22 genes across 15 traits. Next, we used differential expression analysis on a subset of aspen genets with divergent concentrations of salicinoid phenolic glycosides (key defense traits). This complementary method to traditional GWA discovered 1243 differentially expressed genes for a polygenic trait. Soft clustering analysis revealed three gene clusters (241 candidate genes) involved in secondary metabolite biosynthesis and regulation. Our work reveals that ecologically important traits governing higher‐order community‐ and ecosystem‐level attributes of a foundation forest tree species have complex underlying genetic structures and will require methods beyond traditional GWA analyses to unravel.

## Graphical abstract

Our research characterized the genetic architecture of 30 ecologically important traits using a common garden of aspen (*Populus tremuloides*, Michx.) through genomic and transcriptomic analyses. A multilocus association model revealed that most traits displayed a polygenic architecture, with most variation explained by loci with small effects. Our work reveals that ecologically important traits governing higher‐order community‐ and ecosystem‐level attributes of a foundation forest tree species have complex underlying genetic structures.

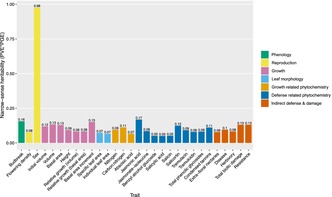

## 1 INTRODUCTION

Ecologically important traits are those that affect an organism's ability to survive and reproduce in natural environments (Stinchcombe & Hoekstra, 2008). Despite the rapid advances in our understanding of the influence of genetic variation on ecologically important traits in non‐model plants and their subsequent influence on ecological processes, the genetic architecture (number of genes, effect sizes, type) underlying those linkages is just beginning to be explored (Crutsinger, 2016; Holliday et al., 2017). Incorporation of genomics into “genes to ecosystems” science could dramatically advance our understanding of fundamental ecological processes, inform predictions of biological plasticity and adaptation to a changing world, and guide efforts toward sustainability of natural and managed ecosystems (Whitham et al., 2008).

In the early 2000s, Whitham and colleagues (Whitham et al., 2006, 2008) proposed a framework to extend genomics to communities and ecosystems. They suggested, then demonstrated, that ecologically important traits of foundation species (such as *Populus* species) could be the bridge connecting underlying tree genes and genomic regions to community and ecosystem structure and function (i.e., extended phenotypes). Extensive research on *Populus* species (e.g., Bailey et al., 2006; Bangert et al., 2006; Madritch et al., 2009; Schweitzer et al., 2004) established that intraspecific variation has heritable effects on associated communities (e.g., herbivorous insects, soil microbes) and ecological processes (e.g., trophic interactions, litter decomposition) at the genotype level, and is largely mediated by plant chemistry. Very few studies, however, have endeavored to identify genes associated with the variation in ecologically important traits that yield extended phenotypes (Crutsinger, 2016).

Given the importance of intraspecific trait variation to tree ecology and forest health, researchers are directing their efforts to understand how genetic and genomic variation influences trait variation within populations (Holliday et al., 2017). Genome‐wide association (GWA) analyses have become the premier strategy for identifying candidate genes associated with variation in traits of interest. Forest trees present formidable challenges to GWA analyses because they are physically large, long‐lived, harbor exceptional genetic diversity, and often have large genomes that are difficult to sequence (Petit & Hampe, 2006). Furthermore, many ecologically important tree traits have complex genetic architectures, often with small allelic effects on the phenotype that are difficult to detect using GWA (Lind et al., 2018).

Human genomics research often pioneers methods, like GWA analyses, that are subsequently used with other organisms (e.g., forest trees) and can be a bellwether for emerging practices in the study of quantitative traits. Traditional GWA studies of highly heritable quantitative traits in humans have revealed relatively few candidate genes with large effects (Robinson et al., 2014; Visscher et al., 2017). As a result, ongoing discussion has focused on where the so‐called “missing heritability” might be found. Although several non‐mutually exclusive explanations have been advanced (Edwards et al., 2014; Génin, 2020; Gibson, 2012; Maher, 2008; Robinson et al., 2014; Young, 2019; Zhou et al., 2022; Zuk et al., 2012), the role of polygenicity (many genes of small to moderate effect influence phenotypic variation) remains substantial. Further analyses of human traits with unexplained heritability have shown that most have a polygenic architecture and that rare variants may play an important (albeit smaller) role in trait variation (Hernandez et al., 2019; Marouli et al., 2017; Visscher et al., 2017; Wood et al., 2014; Yang et al., 2010).

A similar story has been unfolding for forest tree species (Ingvarsson & Street, 2011). Most forest tree GWA studies have identified relatively low numbers of significant loci, explaining a small proportion of variation in highly heritable quantitative traits (Barker et al., 2019; Bresadola et al., 2019; de la Torre et al., 2021, 2022; Fahrenkrog et al., 2017; Hallingbäck et al., 2019; Lind et al., 2018; McKown et al., 2018) with rare exceptions (Wang et al., 2018). Several studies have begun to employ modified and complementary methods to address potential sources of “missing heritability,” following the lead of human genomics research. The multilocus GWA is one extended GWA method that can be used to assess the genomic architecture of potentially polygenic traits (Bresadola et al., 2019; de la Torre et al., 2021). A multilocus GWA provides a way to understand how variation in a marker set is associated with trait variation by evaluating the effects of multiple loci simultaneously on a given phenotype. Additionally, transcriptomic methods such as differential expression are being used to complement GWA methods without the need for extensive species‐specific resources, which are not available for most forest tree species (Carrasco et al., 2017). Our study focuses on aspen (*Populus tremuloides* Michx.), a foundation forest tree species. Aspen is highly genetically diverse (Cole, 2005; Mitton & Grant, 1996) and exhibits very little population structure across its range (Callahan et al., 2013), both ideal characteristics for genome‐wide association analyses. Aspen has the largest geographical range of all tree species in North America and is ecologically important. As a foundation tree species, it provides food and habitat for an estimated 500 plant and animal species, enhances biodiversity, and provides ecosystem services such as carbon sequestration (Madson, 1996; Rogers et al., 2020). At the same time, aspen faces threats from herbivory and climate change (Refsland & Cushman, 2021; Rogers et al., 2020).

Growth and defense traits in aspen show extraordinary variation among genets (Cole et al., 2021; Lindroth et al., 2023). In both natural and controlled environments, aspen has exhibited trade‐offs between growth and defense under variable environmental conditions (Cole et al., 2016, 2021; Cope et al., 2019, 2021; Donaldson et al., 2006; Osier & Lindroth, 2006). For example, salicinoid phenolic glycosides represent a class of secondary metabolites that strongly mediate plant‐herbivore interactions, are negatively associated with growth metrics, and are largely genetically controlled (Boeckler et al., 2011; Cole et al., 2021; Lindroth & St. Clair, 2013; Osier & Lindroth, 2006). Variation in phenolic glycosides influences performance, distribution, and abundance of aspen‐associated herbivores (Donaldson & Lindroth, 2007; Holeski et al., 2016; Lindroth and St. Clair 2013). At the same time, high intraspecific competition has been shown to select fast‐growing and poorly‐defended genotypes (Cope et al., 2021), that could leave some aspen stands vulnerable to pests. How aspen populations respond to environmental pressures has long‐term consequences for intraspecific genetic variation and associated community structure (Barker et al., 2019; Cope et al., 2021). Thus, an understanding of the genetic architecture of intraspecific variation in ecologically important traits (e.g., growth and defense) is critical to their future management and conservation.

Our initial GWA analysis in this system (Barker et al., 2019) was one of the first studies to identify specific genes associated with plant traits that shape herbivore community composition. That work, however, documented fewer than expected associations for highly heritable tree traits. To that end, this current study aimed to explore the genetic architecture underlying phenotypic variation in ecologically important traits using modified GWA and an expanded data set. We aimed to characterize the genomic architecture (number of genes, effects sizes, type) for 30 ecologically important growth and defense traits in aspen using single‐locus and multilocus GWA. We also explored whether high defense‐low growth and low defense‐high growth phenotypes exhibit differential expression patterns. To answer this question, we performed a differential expression analysis on a set of aspen genets, half with extremely high and half with extremely low concentrations of salicinoid phenolic glycosides.

## 2 METHODS

### 2.1 WisAsp common garden

The Wisconsin Aspen (“WisAsp”) common garden was established in 2010 at the Arlington Agricultural Research Station (University of Wisconsin‐Madison) near Arlington, Wisconsin (USA). The source genets were collected along a north–south transect in Wisconsin, USA, (corresponding to the northern subpopulation of aspen, Callahan et al., 2013) and propagated via root cuttings to create the WisAsp common garden. WisAsp contains a total of 1824 *P. tremuloides* trees distributed across four blocks using a randomized complete block design and surrounded by a perimeter of additional aspen trees (*N* = 256). Our data collection occurred during the period of time when trees were 4–7 years old, and the canopy was approaching closure. For detailed information about the garden design and site characteristics see (Barker et al., 2019). Genet identity was verified using microsatellites as described by Cole et al. (2021). A total of 516 unique genets were identified, with an average of 3.51 replicates per genet.

### 2.2 Phenotypic data

Evaluated traits were selected because of their importance to the fitness of aspen (Cole et al., 2021; Cope et al., 2019) and their cascading effects on trophic interactions, community structure, and ecosystem function (Barker et al., 2018; Rubert‐Nason & Lindroth, 2021). Surveys of 30 traits (growth, phenology, reproduction, indirect defense and damage, growth‐ and defense‐related phytochemistry) were carried out at WisAsp common garden each year from 2014 to 2018. Table 1 lists the surveyed traits, provides trait descriptions and specifies the associated publications that contain detailed methodological information.

**TABLE 1:** Description of tree traits surveyed.

Trait	Years data were collected	Description of trait measurement	Units
Phenology			
Budbreak date^b^	2014–2017	Bud break values were obtained using a local 2‐degree polynomial regression to predict when each tree had reached stage three on a 5‐point scale. Then degree day was calculated using a base temperature of 4.4°C and rank transformed to account for year‐to‐year environmental variation	Degree days
Reproduction			
Flowering density^a^	2017–2019	Number of flowering twigs were counted in the early spring each year between 2017 and 2019, representing buds formed during the previous year	Number of flowering twigs
Sex^a^	NA	Sex of each genet determined through genotyping with the TOZ19 marker (Pakull et al., 2015)	NA
Growth			
Initial volume^a^	2012	Volume is calculated as diameter^2^ × height (d^2^h), which is known to correlate well with biomass (Stevens et al., 2007). For 2012, when the trees were too small for a diameter at breast height to be taken, basal diameter (10 cm above ground) was used	cm^3^
Volume^a^	2015–2018	Volume is calculated as diameter^2^ × height (d^2^h), which is known to correlate well with biomass (Stevens et al., 2007). For all four measurement years, diameter at breast height (DBH) was taken at a standard height of 1.4 m	cm^3^
Basal area^a^	2015–2018	Square root (best transform) of area for a given measurement year, π(d/2)^2^ where d is the mean of two orthogonal measurements of diameter. For all four measurement years, diameter at breast height (DBH) was taken at a standard height of 1.4 m	cm^2^
Height^a^	2015–2018	Height of the tallest stem	cm
Relative growth (volume)^b^	2015–2018	Growth represented as the change in volume, expressed as the difference in the natural logarithms of the volumes at two time points	cm^3^
Relative growth (basal area)^a^	2015–2018	Growth as difference in natural logarithms of the basal areas at two time points	cm^2^
Basal area increment^a^	2015–2018	Growth as basal area increment or the difference between the basal areas at two time points	cm^2^
Leaf morphology			
Specific leaf area^a^	2014–2017	Average leaf area divided by dry mass	cm^2^/g
Individual leaf area^a^	2014–2017	Individual average leaf area	cm^2^
Growth‐related phytochemistry			
Nitrogen^a^	2014–2017	Concentration of foliar nitrogen	Percent dry weight
Carbon:nitrogen^a^	2014–2017	Ratio of foliar carbon to foliar nitrogen	NA
Abscisic acid^c^	2017	Concentration of foliar abscisic acid	Percent dry weight
Defense‐related phytochemistry			
Jasmonic acid^c^	2017	Concentration of foliar jasmonic acid	Percent dry weight
Jasmonate isoleucine^c^	2017	Concentration of foliar jasmonate‐isoleucine	Percent dry weight
Benzyl alcohol glucoside^c^	2017	Concentration of foliar benzyl alcohol glucoside	Percent dry weight
Salicylic acid^c^	2017	Concentration of foliar salicylic acid	Percent dry weight
Salicin^a^	2014–2017	Concentration of foliar salicin.	Percent dry weight
Salicortin^a^	2014–2017	Concentration of foliar salicortin.	Percent dry weight
Tremulacin^a^	2014–2017	Concentration of foliar tremulacin.	Percent dry weight
Tremuloidin^a^	2014–2017	Concentration of foliar tremuloidin.	Percent dry weight
Total phenolic glycosides^a^	2014–2017	Sum of salicin, salicortin, tremulacin, and tremuloidin.	Percent dry weight
Condensed tannins^a^	2014–2017	Concentration of foliar condensed tannins.	Percent dry weight
Indirect defense and damage			
Extra‐floral nectaries^a^	2014–2017	Mean number of extrafloral nectaries per leaf, measured using digital scans of collected leaves.	Density/leaf
Disease^a^	2014–2017	Percent of leaf area lost to disease, measured using digital scans of collected leaves and the leaf morphology assessment program Winfolia.	Percent area
Herbivory^a^	2014–2017	Leaf area damaged by leaf scrapers plus holes plus leaf margin removed, measured using digital scans of collected leaves and the leaf morphology assessment program Winfolia.	Percent area
Total biotic damage	2014–2017	Leaf area damaged by both herbivores and disease: Disease + Herbivory.	Percent area
Resistance	2014–2017	100‐total biotic damage.	Percent area

^a^ See Cole et al. (2021) for detailed methodology.

^b^ See Barker et al. (2019) for detailed methodology.

^c^ See Cole et al. (2021) and Boeckler et al. (2013) for detailed methodology.

Detailed collection and processing for the following surveyed traits are described by a previous publication (Cole et al., 2021). The sex of each tree was determined through genotyping a sex‐specific marker, TOZ19 (Pakull et al., 2015). Flowering was assessed by counting the number of flowering twigs per tree. Growth was surveyed by measuring the height and diameter of each tree after each growing season and several growth measures including volume, relative growth, and basal area increment were calculated (Table 1). Foliar morphology and indirect defense/foliar damage were quantified from digital scans of leaves collected each year in late June/early July and early August, respectively. Leaf collection was conducted on each tree by haphazardly selecting four leaves (more if they were small) from one or more branches in each cardinal direction. Finely pulverized freeze‐dried leaves were used to quantify all foliar phytochemicals. Salicinoid phenolic glycoside concentrations (salicin, salicortin, tremulacin, and tremuloidin) were quantified using ultra‐high‐performance liquid chromatography‐mass spectrometry as reported by Rubert‐Nason et al. (2018). Condensed tannin concentrations were quantified colorimetrically using the acid butanol method described by Barker et al. (2019). Nitrogen and carbon values were determined by near‐infrared reflectance spectroscopy and calibrated using reference values from combustion gas chromatography, as outlined by Barker et al. (2019) and Rubert‐Nason et al. (2013).

The following traits were not included in Cole et al. (2021) and are further described here. Concentrations of jasmonic acid, jasmonate‐isoleucine, benzyl alcohol glucoside, and salicylic acid were quantified by ultra‐high‐performance liquid chromatography, using the methods outlined by Boeckler et al. (2013). These phytochemical analyses were performed using finely pulverized freeze‐dried leaves. Phenology was assessed by recording the timing of bud break every 2–3 days in the spring using a 5‐point scale as described by Barker et al. (2019). Bud break values were obtained using a local 2‐degree polynomial regression adapted from Rohde et al. (2011), where bud break stage (e.g., 1–5 on the point scale) was the response and the dates of observation were the predictors, to generate a prediction equation for the date each tree reached stage three. Only predicted values with an *R* ^2^ ≥ .88 were kept.

### 2.3 Genomic data

Exome sequence data for 506 genets were obtained via sequence capture genotyping and the sequence data were aligned to the *Populus tremula* v1.1. genome (Lin et al., 2018). Complete details on how the sequencing and alignment were performed for those 506 genets can be found in Barker et al. (2019) and File S1. Subsequently, genet verification via microsatellite analysis revealed that 10 genets had no sequence data. Those genets, plus an additional 15 genets that were omitted by Barker et al. (2019) because of poor‐quality sequencing, were sent for sequence capture genotyping using the same probes and methodology as the original 506 sequenced genets. Additionally, a subset of genets (*N* = 11) that had been sequenced previously with whole‐genome sequencing (for complete details, see Wang et al. (2016)) were included because many of them had better quality sequencing data than when sequenced using sequence capture genotyping. A joint call over all samples (*N* = 506 + 25 + 11 = 542) was conducted using GATK GenotypeGVCFs with a standard emit confidence of 10 and a standard call confidence of 20 resulting in the discovery of 6,827,282 SNPs. Variants were filtered for genotype quality and sample quality metrics using VCF and BCF tools (Danecek et al., 2011). The full variant filtering pipeline is provided in File S1. Duplicate samples (including clones) were removed by keeping the sample with the best quality sequencing data (*N* = 53 duplicate samples removed). An additional 32 samples were removed for poor sequence quality (>20% missing data) and two were removed because they were likely F1 hybrids of *P. tremuloides* and *grandidentata* (File S2). After variant filtering, missing genotype information was imputed using LinkImpute (Money et al., 2015). The SNP filtering pipeline resulted in a data set of 455 replicated genets with 291,069 SNPs distributed across 5375 scaffolds and 20,875 genes with 10 SNPs per gene on average.

To assess the presence of population structure, we used ADMIXTURE (Alexander et al., 2009), which employed maximum likelihood estimation of individual ancestries based on our SNP data to determine the most likely number of populations or distinct groups present in the sample set. We tested values 1 through 5 for *k* (i.e., the number of groups) and used cross‐validation to validate the results. ADMIXTURE estimated that our samples most likely represent a single population, with high confidence (See File S1 for full ADMIXTURE analysis details). Five pairs of genets exhibited sibling or parent‐offspring relationships (see File S1). These genets were not excluded to maximize sample size and because an initial GWA run with and without the sibling pairs excluded showed no difference in results (data not presented). Additionally, as presented above, ADMIXTURE did not identify any population structure with the five sibling pairs included. File S2 contains further details about all sequenced samples and excluded samples.

The exome capture sequencing data were aligned to the *Populus tremula* genome assembly v1.1 (Potra v1.1) because that was the best available assembly at the time of the sequencing data processing. During the analysis of the GWA results, the most recent assembly, *Populus tremula* genome assembly v2.2 (Potra v2.2) became available. In order to be able to estimate where candidate genes might be located within the genome, we developed an ad hoc method to connect the Potra v1.1 genes to the Potra v2.2 genes. We performed a reciprocal BLAST on gene lists from both assemblies. We matched only genes with a sum of ranks equal to zero (i.e., the genes were each other's best match in both assemblies).

### 2.4 Transcriptomic data

We used an extreme phenotyping sampling scheme to select genets from the WisAsp common garden for total RNA sequencing. Using foliar salicinoid phenolic glycoside (PG) concentrations from June 2016, 30 genets with high constitutive PG concentrations (10%–16% leaf dry mass) and 30 genets with low constitutive PG concentrations (1%–2.5%) were sampled for a total of 60 genets. Leaf samples from a minimum of four replicate trees per genet were collected on 1 day in July 2017 (6–7 year‐old trees). Extraction of total RNA was performed using the RNeasy Plant Mini Kit with DNase digestion (Qiagen, Valencia, CA), and quality control and quantification were performed on the Agilent 2100 BioAnalyzer (Agilent Technologies, Santa Clara, CA) at the University of Wisconsin Biotechnology Center (Madison, WI, USA). Samples were sent to the Michigan State University RTSF Genomics Core (East Lansing, MI, USA) for RNA library preparation and sequencing. TruSeq stranded mRNA libraries were divided into three pools and each run in a separate lane of an Illumina HiSeq4000 flow cell (San Diego, CA, USA) in 2 × 150 bp paired‐end mode to an average depth of 16 million read pairs per sample.

Raw RNA sequence data were put through a standard filtering pipeline. Ribosomal RNA removal was completed using default settings in SortMeRNA (Kopylova et al., 2012) to reduce rRNA alignment bias. Trimmomatic (Bolger et al., 2014) was used to remove partial adaptor sequences within the sequenced reads and to perform quality‐based trimming. Quality‐based trimming works by “trimming” low‐quality bases from the 3′ end until the quality reaches a specified Phred‐quality threshold. We used a standard Phred‐quality threshold of 20 corresponding to a base call error of 1 in 100, which is approximately the inherent technical error rate of the Illumina sequencing platform. FASTQC (Andrews, 2010) and multiqc (Ewels et al., 2016), were used after each filtering step to assess sample quality (e.g., per base sequence quality, per sequence quality scores, per base sequence content, and per sequence GC content). The filtered RNA sequences were then quasi‐aligned to the *Populus tremula* v.2.2 transcriptome (see Schiffthaler et al. (2019); assembly files are available through the FTP site at https://plantgenie.org/) using the default k‐mer of 31 (optimized for reads ≥75 bp) and quantified using Salmon (Patro et al., 2017). Quasi‐alignment can reduce computational time and provide a way to align and quantify transcripts for organisms with limited genomic resources. As performed in Salmon, it uses a reference index created from a given reference transcriptome by evaluating the sequences for all possible unique sequences of length k (k‐mer) in the transcriptome. That reference index is then used to estimate where the raw sequencing reads best align without performing base‐by‐base alignment, decreasing computational time substantially. Then the final transcript abundance estimates are generated after modeling sample‐specific biases (e.g., GC and sequence biases), which, if not accounted for, are known to create high false positive rates in differential expression. File S3 contains a detailed description of the RNA filtering and quality assessment pipeline.

### 2.5 Genome‐wide association analyses

The first step of our genome‐wide association analyses was fitting the linear mixed model shown in Equation 1 to each trait in order to extract the genotypic mean effect for each genet across all replicates (3.51 replicates per genet on average). In this formulation, *x*_*ijky*_ is the trait value during year *y* for individual *i* belonging to genet *j* and residing in block *k*, and *μ* is the grand mean of the trait. The independent variables in the model include block, perimeter membership ($p_{i}$), age at the time of sampling ($a_{\mathrm{iy}}$), initial size of the tree ($s_{i}$), and their fixed effects α, $\beta_{1}$, $\beta_{2}$, and $\beta_{3}$, respectively. The trait value also depends upon random effects for genet ($\gamma_{j}$) and year ($\lambda_{y}$). The random effects and the random error term ($\varepsilon_{\text{ijky}}$) all follow a zero‐mean Gaussian distribution. These models were fit using the lme4 R package (Bates et al., 2015).

(1) $$\begin{array}{c} x_{\text{ijky}}=\mu +\alpha_{k}+\beta_{1}p_{i}+\beta_{2}a_{\mathrm{iy}}+\beta_{3}s_{i}+\gamma_{j}+\lambda_{y}+\varepsilon_{\text{ijky}}\\ \gamma_{j}∼N\left(0,\sigma_{\gamma }^{2}\right)\lambda_{y}∼N\left(0,\sigma_{\lambda }^{2}\right)\varepsilon_{\text{ijky}}∼N\left(0,\sigma^{2}\right) \end{array}$$

Block and perimeter positions were included to control for microenvironmental differences and edge effects. Tree age and initial size were included to account for replanting of a quarter of the ramets in 2011 and 2012 due to vole damage (Cole et al., 2021). A subset of tree traits (salicylic acid, jasmonic acid, jasmonate‐isoleucine, abscisic acid, benzyl alcohol glucoside) was collected at only one time point, so survey year was not included in the model for those traits. Phenotypic trait data were transformed to meet normality assumptions of linear mixed models as needed and *z*‐scale was normalized to standardize effect sizes among tree traits. From these models, the estimated best linear unbiased predictors (BLUP) of genet effects ($\gamma_{j}$) were extracted. These BLUP values represent the average effect of each genet across replicates on a trait that is attributable to genetic factors. These values were then rank transformed, which has been shown to improve the sensitivity of GWA analyses when sample sizes or genetic effects are small (Goh & Yap, 2009), and regressed on each genetic variant (i.e., GWA).

Broad‐sense heritabilities were calculated by dividing the genet‐associated variance component ($\sigma_{\gamma }^{2}$) by the total variance ($\sigma_{x}^{2}$) as shown in Equation 2.

(2) $$H^{2}=\frac{\sigma_{\gamma }^{2}}{\sigma_{x}^{2}}$$

Variance components were extracted using the VarCorr function from the R package lme4 (Bates et al., 2015). Since the linear mixed model includes repeated measurements for the same individual (i.e., multiple survey years), we calculated the correlation between repeated measurements of the same individual (i.e., repeatability) to assess the accuracy of our broad‐sense heritability estimates (Figure S1) (Falconer & Mackay, 1996). Tables S1 and S2 display model characteristics and variance components for each trait, respectively. All analyses were performed in R v. 3.4.4, 3.5.1, and 3.6.1 (R Core Team, 2018).

A multilocus GWA provides a way to understand how variation in a marker set is associated with trait variation. It does so by modeling how much of that trait variation is likely explained by loci with relatively larger effects as compared to the infinitesimal effects of all loci. In short, it models the genetic architecture of the trait. GEMMA's Bayesian Sparse Linear Mixed Model (BSLMM) (Zhou et al., 2013) combines a ridge regression (i.e., models relatively small effect variants) and a Bayesian variable sparse regression (i.e., models relatively large effect variants) to associate phenotypes and loci by modeling all variants simultaneously. The model estimates the amount of phenotypic variance explained by sparse and random effects, defined as PVE, where sparse effects are relatively large effect loci and the random effects are the relatively small effect loci. The estimated value of the sparse effects is defined as PGE or proportion of genetic variance and indicates what proportion of PVE is explained by loci with relatively large effects. Multiplying PVE by PGE provides an estimate of the proportion of total phenotypic variance explained by the sparse effects (i.e., loci with large effect sizes relative to all the loci included in the model) for each trait, also known as narrow‐sense heritability, h^2^ (Bresadola et al., 2019). The model also generates an estimate of the putative number of sparse effect loci (e.g., loci with large effect sizes relative to all the loci included in the model) called n_gamma, which can provide further context for the genetic architecture of a trait of interest in combination with the PVE and PGE values. Each trait was run through the BSLMM for 10 runs with a two million burn‐in followed by 10 million iterations (script available in File S4). Values for PVE and PGE were evaluated across all 10 runs for consistency (Figure S2) and one representative run was chosen for data presentation.

All GWA analyses were carried out in Plink 1.9 which performs simple linear regression using the Wald statistic to generate *p*‐values (scripts available in File S4). We did not correct for population structure as our population was panmictic (Barker et al., 2019). Traditional single‐locus GWA was performed for each of the 30 traits. Many of our growth and defense traits are likely functionally related (Cole et al., 2021; Cope et al., 2019, 2021) and significant genetic correlations were shown for many of them (using Pearson's correlation coefficient, Figure S3). Genetically correlated traits (and even functionally related traits in the absence of genetic correlations) can be analyzed simultaneously to improve the power of GWA (Chhetri et al., 2019; Stephens, 2013). We conducted multi‐trait GWA for growth and defense traits with genetic correlations or functional relationships (for a full list of trait combinations see File S5). Multiple testing was accounted for by applying a false discovery rate correction, specifically, Storey's q‐value (Storey & Tibshirani, 2003) using a threshold of 0.2. Unlike stringent Bonferroni‐based multiple testing corrections, Storey's q‐value corrects for false positives, while reducing the number of false negatives.

### 2.6 Transcriptomic analyses

To identify any confounding factors such as batch effects and to determine if conditions were sufficiently separated, quality assessment of the count data was performed in R v. 3.6.1 (R Core Team, 2018) as detailed in File S3. Differential expression was carried out in DESeq2 (Love et al., 2014), which fits a negative binomial generalized linear model and automatically normalizes counts by library size. We used an adjusted *p*‐value cut‐off of .05 and a log fold change cut‐off of 0 given our large sample size, following recommendations by Schurch et al. (2016). We analyzed the differentially expressed genes through the application of a soft clustering method in the function Mfuzz that uses a fuzzy c‐means algorithm (Futschik & Carlisle, 2005; Kumar et al., 2007). Soft clustering using Mfuzz is more robust to noise than hard clustering methods because it allows genes to be a member of more than one cluster, thus providing a measure of how well corresponding clusters represent gene expression patterns. This attribute allows users to make nuanced inferences about the role genes may play in different functional clusters. Additionally, because the method minimizes the variation of genes within a cluster, genes that poorly cluster will have less influence on a cluster and thereby make the clustering process less sensitive to noise. This attribute is beneficial because no genes were filtered out to reduce noise, enabling us to keep potentially important information. Clustering parameters were set using the methodology outlined by Schwämmle and Jensen (2010). Only genes with a membership of ≥0.75 were kept in each cluster. A gene enrichment analysis was performed for each cluster of genes against the background of all *Populus tremula* (v2.2) genes using the enrichment analysis tool from PlantGenIE (https://plantgenie.org/).

## 3 RESULTS

### 3.1 Genetic architecture of tree traits

#### 3.1.1 Broad‐sense heritabilities

**FIGURE 1:**
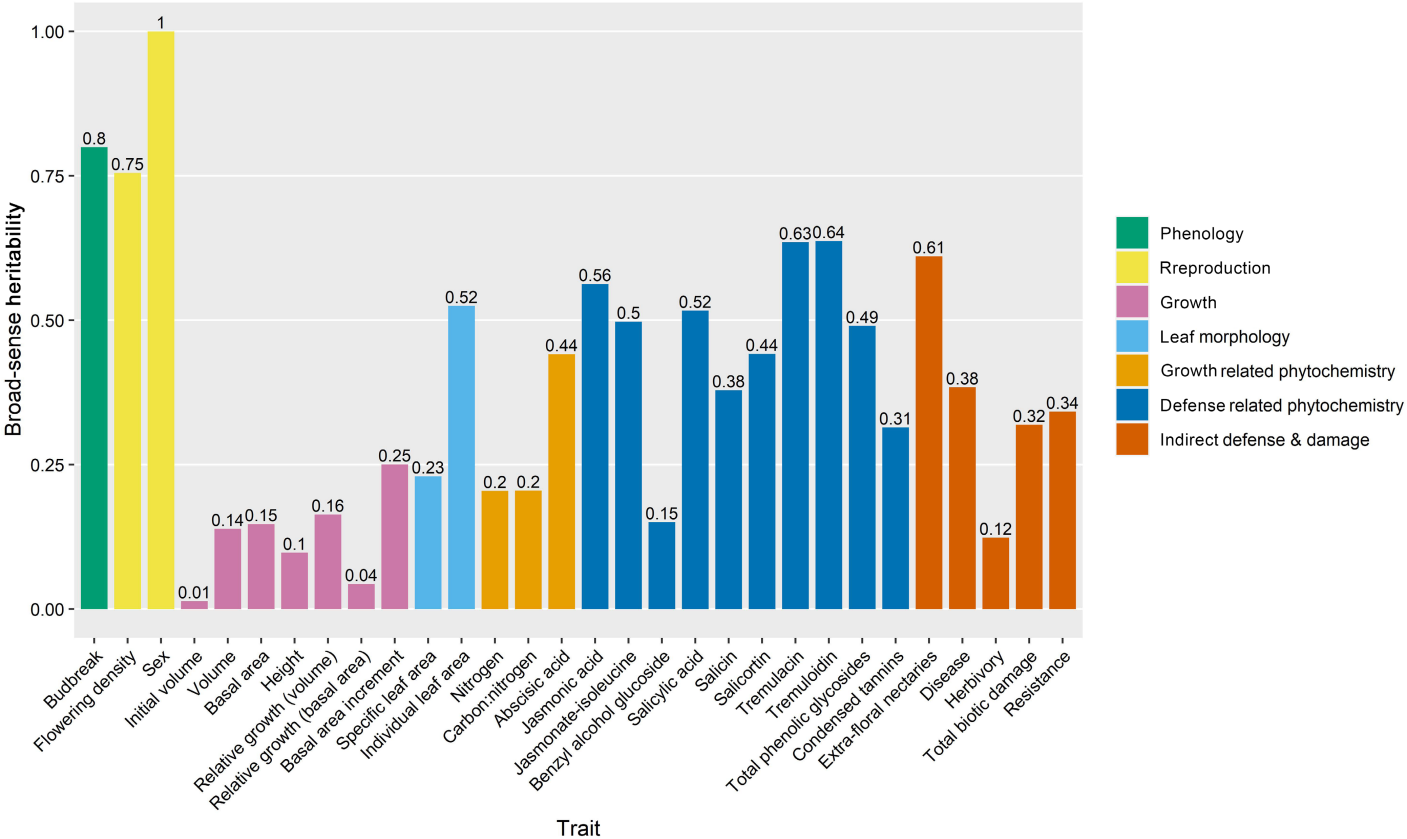
Broad‐sense heritabilities for ecologically important traits in aspen (*Populus tremuloides*). Values calculated by dividing the genet‐associated variance component by the total random variance component extracted from linear mixed models (variance components in Table S1).

We calculated broad‐sense heritabilities for all of our tree traits to understand what proportion of the variation was explained by genet identity alone and to compare to the narrow‐sense heritability estimates obtained from our multilocus GWA. Sex can be considered as a pseudo‐control trait as it would be expected to exhibit a broad‐sense heritability at or approaching 1 given that it is a genetically controlled trait with limited environmental influence and a known genetic architecture (Müller et al., 2020). Budbreak date displayed a high level of heritability (H^2^ = 0.80) expected from a highly genetically controlled trait (Frewen et al., 2000). Flowering density was moderately heritable (H^2^ = 0.37). It should be noted that only about a third of the trees at WisAsp had reached reproductive maturity at the time of this study, so the sample size was smaller than for the other traits. In general, traits associated with defense (defense‐related phytochemistry and indirect defense) had higher heritability than growth traits (growth, growth‐related phytochemistry, and leaf morphology) (Figure 1). The broad‐sense heritabilities were moderate to high (H^2^ = 0.15–0.64) for most defense traits and low to moderate (H^2^ = 0.01–0.52) for most growth traits. Diseased tissue showed a moderate level of heritability (H^2^ = 0.38), while herbivory (i.e., foliar tissue damaged by insects that scrape and/or remove leaf area) exhibited low heritability (H^2^ = 0.12). Total biotic damage and resistance (1‐total biotic damage), which include area damaged by both disease and herbivory, also showed moderate levels of heritability (0.32 and 0.34 respectively).

#### 3.1.2 Multilocus GWA results

**FIGURE 2:**
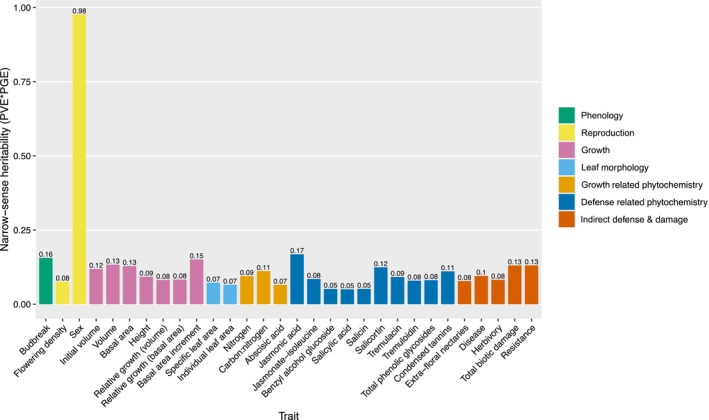
Narrow‐sense heritabilities. Values calculated by multiplying PVE by PGE (Bresadola et al., 2019) estimates generated by GEMMA's BSLMM model; PVE = proportion of phenotypic variance explained by sparse and random effects, PGE = proportion of phenotypic variation explained by sparse effects only (i.e., relatively large effect loci).

The narrow‐sense heritabilities (h^2^) displayed in Figure 2 give an overview of the genetic architecture of each trait. For the majority of tree traits, our SNP dataset explained very little variation, excluding sex (mean = 0.10, median = 0.09, range = 0.05–0.17). In other words, most of the phenotypic variation for a particular trait is likely explained by many loci with relatively small effects, indicative of a polygenic architecture. Budbreak date, a typically highly heritable trait in *Populus* (Frewen et al., 2000), had a lower narrow‐sense heritability (h^2^ = 0.16) than expected, but still higher than the majority of other traits. Narrow‐sense heritability for sex, a highly genetically controlled trait, explains phenotypic variation at close to expected values (i.e., broad‐sense heritability).

**FIGURE 3:**
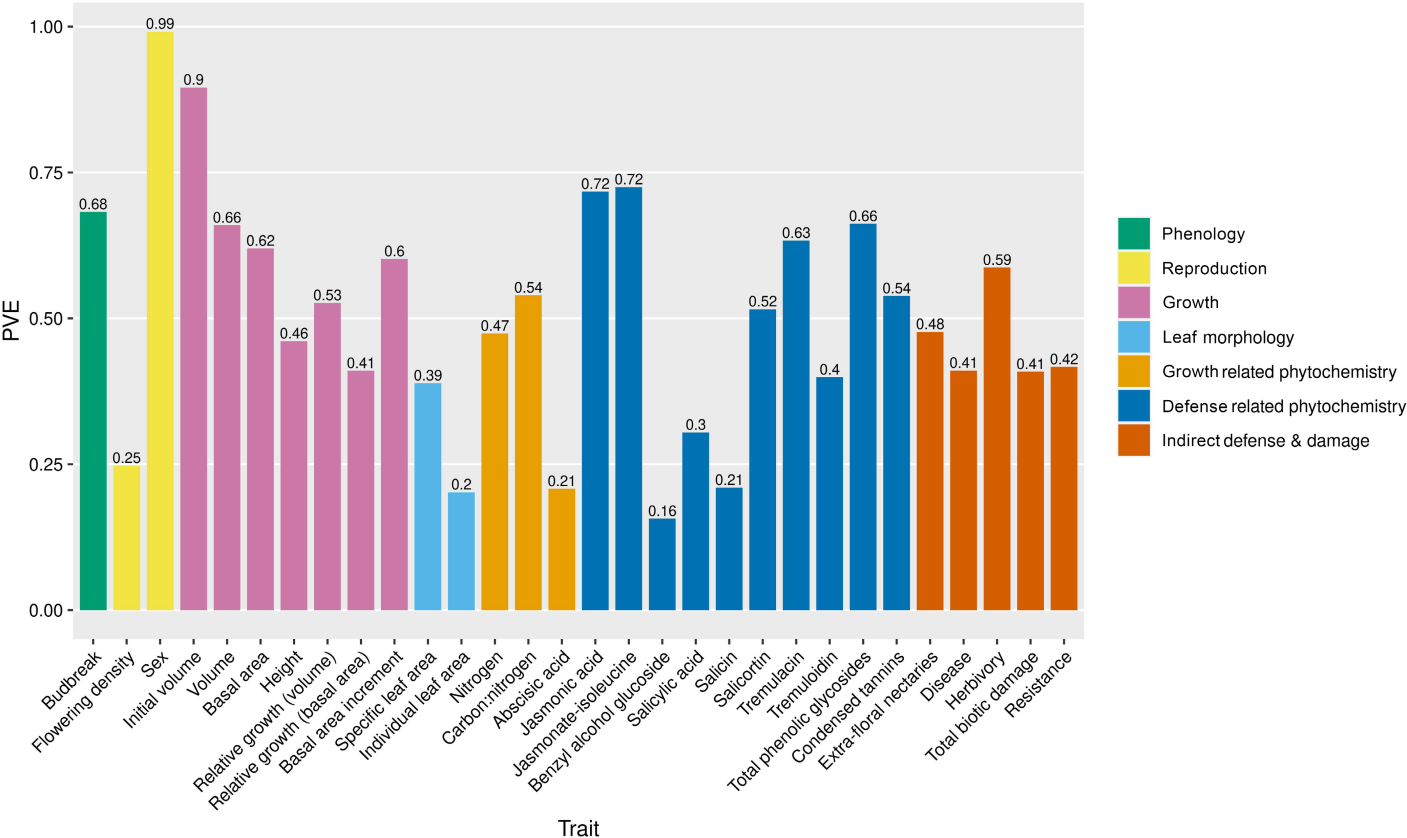
Hyperparameter PVE from GEMMA's BSLMM model; PVE = proportion of phenotypic variance explained by sparse and random effects (relatively large and small effect loci).

The narrow‐sense heritabilities (h^2^) are calculated from PVE and PGE estimates, values that can provide a more nuanced view of trait genetic architecture (Figure 3 and Figure S4). PVE is the proportion of phenotypic variation explained by all loci and PGE is the proportion of PVE explained by loci with relatively large effects. The proportion of relatively large effect loci (PGE) is less than ~0.40 for all traits except sex (mean = 0.26; range 0.14–0.41). This pattern holds true for traits such as initial volume and jasmonate‐isoleucine, where our marker set explains a large amount of phenotypic variation (PVE > 0.70) (Figure 3). Posterior probability density distributions indicate that PGE values for the majority of loci are less than 0.25 across almost all traits (Figure S5). Thus, for many of our traits, most of the loci affecting trait variation likely have relatively small effects (random effects portion of PVE). Consistent with this finding, estimated effect sizes were extremely low for most loci across all traits (Table 2).

**TABLE 2:** Effect sizes for multi‐SNP GWA.

Trait	Median sparse effect size	Mean sparse effect size	n_gamma
Budbreak	0.000004	0.00003	70
Flowering density	0.000002	0.000009	58
Sex	0	0.00002	18
Initial volume	0.000004	0.00002	87
Volume	0.000003	0.00001	132
Basal area	0.000004	0.00002	90
Height	0.000003	0.000009	97
Relative growth (volume)	0.000002	0.000009	98
Relative growth (basal area)	0.000003	0.000009	97
Basal area increment	0.000005	0.00002	98
Specific leaf area	0.000002	0.000008	54
Individual leaf area	0.000002	0.000006	89
Nitrogen	0.000003	0.00001	68
Carbon:nitrogen	0.000004	0.00001	75
Abscisic acid	0.000003	0.00001	59
Jasmonic acid	0.000005	0.00002	75
Jasmonate‐isoleucine	0.000004	0.00001	59
Benzyl alcohol glucoside	0.000002	0.000007	68
Salicylic acid	0.000003	0.00001	41
Salicin	0.000002	0.000008	57
Salicortin	0.000004	0.00001	111
Tremulacin	0.000005	0.00002	60
Tremuloidin	0.000004	0.00001	69
Total phenolic glycosides	0.000004	0.00001	48
Condensed tannins	0.000002	0.00009	104
Extra‐floral nectaries	0.000005	0.00002	58
Disease	0.000002	0.00001	50
Herbivory	0.000004	0.00001	88
Total biotic damage	0.000004	0.00002	25
Resistance	0.000003	0.00001	42

Sex shows that PVE and PGE are both high, approaching 1 (i.e., 99% phenotypic variance explained by genomic data, with 98% of that variance attributable to relatively large effect loci). Sex also has the lowest n_gamma value of all the traits (n_gamma = 17) (Table 2). Together, these estimates reveal that the genetic architecture for sex is highly heritable underlain by a few loci with relatively large effects, consistent with its known genetic architecture (Müller et al., 2020). Traits like budbreak date and jasmonic acid display a highly heritable genetic architecture (PVE > 0.60) where some loci of relatively large effect (i.e., PGE > 25%) exist within a polygenetic background. Both budbreak date and jasmonic acid have relatively high n_gamma values of 70 and 75, respectively (Table 2). In contrast, traits such as total phenolic glycosides and initial volume also have a highly heritable genetic architecture (PVE > 0.60) but are largely explained by relatively small effect loci (i.e., PGE < 15%). N_gamma values are 48 and 87 for phenolic glycosides and initial volume, respectively (Table 2). All multilocus GWA parameters and corresponding estimates for each trait can be found in File S6.

### 3.2 Candidate genes: Single‐locus GWA results

**FIGURE 4:**
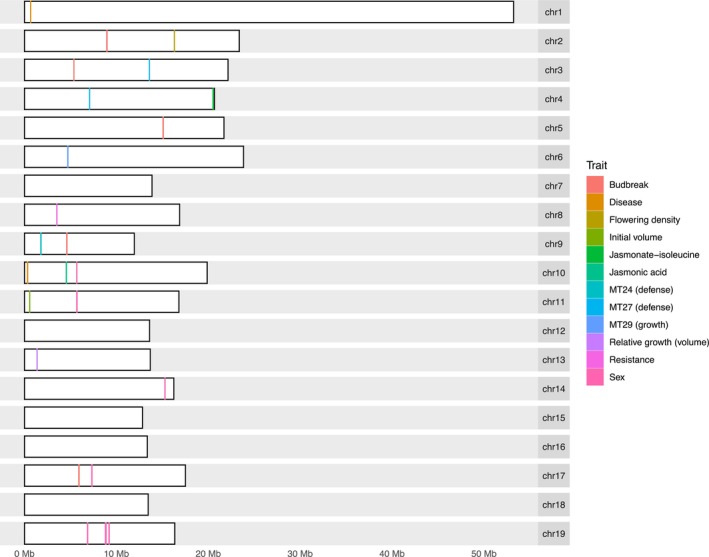
Distribution of candidate genes across *Populus tremula* genome assembly v2.2 using a reciprocal BLAST to match Potra v1.1 genes to Potra v2.2 genes (see Methods for details); chromosomes are indicated by the white bars on a gray background; colored vertical lines represent genes associated with significant SNPs, with different colors corresponding with the specific trait associated with the significant SNP as notated in the legend.

Our single‐locus genome‐wide association analyses used a marker data set of 291,069 SNPs and found 38 significant SNPs in 22 genes across 15 traits. Budbreak date accounted for six of the 22 genes identified (Table 3). An additional 40 SNPs and 15 genes were associated with sex. One candidate gene was associated with flowering density. Growth traits were associated with four genes. Defense‐related phytochemical traits were associated with eight genes. Indirect defense and damage (i.e., total biotic damage, diseased foliar tissue, and herbivory) accounted for six identified genes. More detailed information for each candidate gene can be found in Files S5 and S7. Except for sex, candidate genes for any particular trait were spread across the genome (Figure 4). Genomic positional annotation through snpEff found most of the candidate genes having regulatory roles (Table 3). Effect sizes of the significant SNPs for most traits were low to moderate (β < ±0.4) excluding sex (File S7). Minor allele frequencies were also low to moderate (0.005–0.3) excluding budbreak date and sex (File S7).

**TABLE 3:** Summary table for single‐locus genome‐wide association analysis results (See Files S5 [multi‐trait GWA] and File S8 [univariate GWA] for more details).

Trait	Gene ID and description (using Potra v1.1 assembly on popgenie.org)	GO annotation(s)	SNPeff annotation(s) (number of SNPs)
Phenology			
Budbreak date	Potra003338g21388: DNA repair protein REV1 isoform X1	Damaged DNA binding, nucleotidyltransferase activity, error‐prone translesion synthesis	Synonymous gene variant (1), missense gene variant (1)
Budbreak date	Potra000831g06663: pentatricopeptide repeat‐containing protein At1g71490	Chloroplast thylakoid membrane	Synonymous gene variant (3), missense gene variant (2), splice region variant & intron variant (2), stop lost (1), 3′ UTR variant (1)
Budbreak date	Potra002372g18071: transcription factor MYB108‐like	DNA binding	5′ UTR variant (1)
Budbreak date	Potra003956g23750: ABC transporter B family member 15‐like	ATP binding, integral component of membrane, ATPase activity, coupled to transmembrane movement of substances, transmembrane transport	5′ UTR variant (1)
Budbreak date	Potra003186g20936: transmembrane protein 53	Integral component of membrane, hydrolase activity	Downstream gene variant (4)
Budbreak date	Potra000419g02166: membrane‐bound transcription factor site‐2 protease homolog isoform X1	Metalloendopeptidase activity, proteolysis, membrane	3′ UTR variant (1)
Reproduction			
Flower density	Potra004072g24439: O‐fucosyltransferase family protein	Cytoplasm, fucose metabolic process, transferase activity, transferring glycosyl groups	5′ UTR variant (1)
Growth			
Relative growth (volume) between 2016 and 2017	Potra000177g00680: probable polyol transporter 4	Integral component of membrane, transmembrane transporter activity, transmembrane transport	Synonymous variant (1)
Initial volume	Potra002519g19030: chromosome condensation regulator family protein	Metal ion binding	Intron variant (1)
Initial volume	Potra000790g06258: tubulin beta chain	GTPase activity, structural constituent of cytoskeleton, GTP binding, microtubule, microtubule‐based process	Upstream gene variant (1)
Growth, leaf morphology, growth‐related phytochemistry			
MT29: individual leaf area, specific leaf area, volume, nitrogen	Potra001566g12942: circadian locomoter output cycles protein kaput isoform X1	Protein binding	Synonymous variant (1)
Defense‐related phytochemistry			
Jasmonic acid	Potra000530g03683: cation/H(+) antiporter 15‐like	Cation transport, solute:proton antiporter activity, integral component of membrane, transmembrane transport	Synonymous variant (1)
Jasmonate‐isoleucine	Potra002923g20319: long‐chain‐alcohol oxidase FAO4A	Long‐chain‐alcohol oxidase activity, flavin adenine dinucleotide binding, oxidation–reduction process	Missense variant (1)
Indirect defense & damage			
Disease	Potra001342g11489: DNA‐dependent metalloprotease WSS1‐like	NA	Upstream gene variant (1)
Total biotic damage Disease Resistance	Potra004005g24127: F‐box/kelch‐repeat protein At1g55270	Protein binding	Downstream gene variant (1)
Resistance	Potra000464g02731: GATA zinc finger domain‐containing protein 8‐like	NA	Downstream gene variant (1)
Defense‐related phytochemistry, indirect defense & damage			
MT27: salicin, salicortin, tremulacin, tremuloidin	Potra002739g19881: nudix hydrolase 18, mitochondrial‐like	Hydrolase activity	5′ UTR variant (2)
MT27: salicin, salicortin, tremulacin, tremuloidin	Potra003968g23830: gibberellin 3‐beta‐dioxygenase 1‐like	Oxidoreductase activity, oxidation–reduction process	Intron variant (1)
MT27: salicin, salicortin, tremulacin, tremuloidin	Potra002382g18137: serine/threonine‐protein kinase STY8 isoform X1	Protein kinase activity, ATP binding, protein phosphorylation	Intron variant (1)
MT27: salicin, salicortin, tremulacin, tremuloidin	Potra001164g10107: serine/threonine‐protein kinase HT1‐like	Protein kinase activity, ATP binding, protein phosphorylation	Intron variant (1)
MT27: salicin, salicortin, tremulacin, tremuloidin	Potra001654g13571: leucine‐rich repeat extensin‐like protein 4	Protein binding	Missense variant (1)
MT10: benzyl alcohol glucoside, total biotic damage, salicylic acid MT30: jasmonic acid, abscisic acid, salicylic acid	Potra004005g24127: F‐box: /kelch‐repeat protein At1g55270	Protein binding	Downstream gene variant (1)
MT23: disease, jasmonic acid, specific leaf area, budbreak date, herbivory MT24: herbivory, salicylic acid, disease, total phenolic glycosides	Potra004005g24127: F‐box: /kelch‐repeat protein At1g55270	Protein binding	Downstream gene variant (1)
MT24: herbivory, salicylic acid, disease, total phenolic glycosides	Potra001342g11489: DNA‐dependent metalloprotease WSS1‐like	NA	Upstream gene variant (1)
MT24: herbivory, salicylic acid, disease, total phenolic glycosides	Potra000572g04244: expansin‐like A2	Extracellular region, sexual reproduction	Missense variant (1)

Budbreak date exhibited the highest number of significant SNPs for a single trait excluding sex. Nineteen SNPs across six genes were associated with budbreak date. A notable gene encodes for a pentatricopeptide repeat‐containing protein (PPR). Proteins with PPR regulate the expression of genes involved in organelle biogenesis and have an impact on plant growth and development (Barkan & Small, 2014). Another gene, encoding a MYB108 transcription factor, is potentially involved in phenological maturation, chloroplast development, and response to abiotic/biotic stress in *Arabidopsis* (Ambawat et al., 2013; Mandaokar & Browse, 2009; Zhao et al., 2020). Three other candidate genes have potential roles in transport across the chloroplast thylakoid membrane, chlorophyll biogenesis (Adam, 2013), and transporting hormones and other compounds essential for plant growth and development across various biological membranes (Hwang et al., 2016; Martinoia et al., 2002). The remaining two candidate genes for budbreak date have not been studied well or at all in plants to date.

As expected, the sex‐determining gene ARR‐17 (Bräutigam et al., 2017; Müller et al., 2020) was discovered as one of the genes with a significant SNP for the sex trait. For sex, the majority of significant SNPs and associated genes were located on chromosome 19, which is thought to be where the sex‐determining region is located for *Populus* species. These GWA results are consistent with the genetic architecture suggested by our multilocus GWA, highly heritable with a few loci contributing to the majority of the phenotypic variation (see Figures 2 and 3). Flowering density exhibited a single association, located within an O‐fucosyltransferase family protein gene, which is known to be involved in the regulation of the plant circadian clock (Liu & Gendron, 2020; Zentella et al., 2017).

Few significant associations were found for growth and growth‐related phytochemical traits, consistent with their narrow‐sense heritabilities being generally low (Figure 2). Univariate GWA found three significant SNPs in three genes, all associated with volume metrics. A tubulin beta chain gene and a probable polyol transporter are involved in morphogenesis (Snustad et al., 1992) and sugar transport (Johnson et al., 2006), respectively. The third gene encodes a chromosome condensation regulator family protein, with no known direct connections to plant growth. Multi‐trait GWA discovered only one association for multi‐trait group MT29 including individual and specific leaf area, volume, and nitrogen concentration. The SNP was located in a circadian clock regulatory gene, with known function in animals, but not well‐defined in plants (Liu & Gendron, 2020).

For defense‐related phytochemical traits, only the plant hormone jasmonic acid and its derivative jasmonate‐isoleucine have significant associations in the univariate GWA, consistent with the PGE estimate suggesting a genetic architecture of being polygenic with some relatively large effect loci. The candidate genes were a cation/H(+) antiporter gene and a long‐chain‐alcohol oxidase FAO4A gene. A transcriptome analysis in switchgrass revealed that a vacuolar Na+(K+)/H+ antiporter gene upregulated JA when overexpressed (Huang et al., 2018). There is little functional information for the gene encoding a long‐chain‐alcohol oxidase FAO4A, making its association with jasmonate‐isoleucine unclear at this time. Multi‐trait GWA identified a F‐box/kelch‐repeat protein associated with two defense‐related multi‐trait groups, including MT10 (benzyl alcohol glucoside, total biotic damage, salicylic acid) and MT30 (abscisic acid, jasmonic acid, salicylic acid). A multi‐trait GWA including all four salicinoid phenolic glycoside constituents (multi‐trait group MT27) resulted in six significant SNPs within five genes. Two genes encode serine/threonine‐protein kinases, which are known to be involved in the regulation of plant defense (Afzal et al., 2008; Hardie, 1999). Another two encode genes that seem more related to growth than to defense. One encodes a gibberellin 3‐beta‐dioxygenase, which may be involved in the negative regulation of jasmonate to repress defense response and promote growth (Bhattacharya et al., 2012). The other encodes a leucine‐rich repeat extensin‐like protein that is involved in cell wall sensing indirectly relaying extracellular signals, including biotic stimuli, to the cytoplasm (Herger et al., 2019). The last gene encodes a nudix hydrolase belonging to a large family of genes that help regulate diverse biological processes through cytosolic and organellar housecleaning and maintain physiological homeostasis (Huang et al., 2012; Ogawa et al., 2008).

Four SNPs within three genes were associated with indirect measures of defense and damage (i.e., disease, total biotic damage, and resistance). The F‐box/kelch‐repeat protein associated with defense traits was also associated with disease, total biotic damage, and resistance. A metalloprotease WSS1‐like gene was associated with disease and is involved in DNA‐protein crosslink repair, damage that often happens due to reactive oxygen species in the cell during regular metabolic processes or in response to a pathogen attack (Enderle et al., 2019). Finally, a GATA zinc finger domain‐containing protein like‐8 was associated with resistance. This specific gene has not been characterized well, but studies characterizing families of genes with GATA zinc finger domains have connected them to circadian clock regulation and plant development in *Arabidopsis* and rice (Behringer & Schwechheimer, 2015; Reyes et al., 2004), and a variety of potential regulatory roles in *Populus* including circadian clock, phytohormone, plant development, and stress response (An et al., 2020). Multi‐trait GWA resulted in only one new association for multi‐trait group MT24 (herbivory, salicylic acid, disease, total phenolic glycosides), located in an expansin‐like A2 gene potentially involved in growth and defense (Abuqamar et al., 2013; Yang et al., 2014).

### 3.3 Differential expression and soft cluster analyses

**FIGURE 5:**
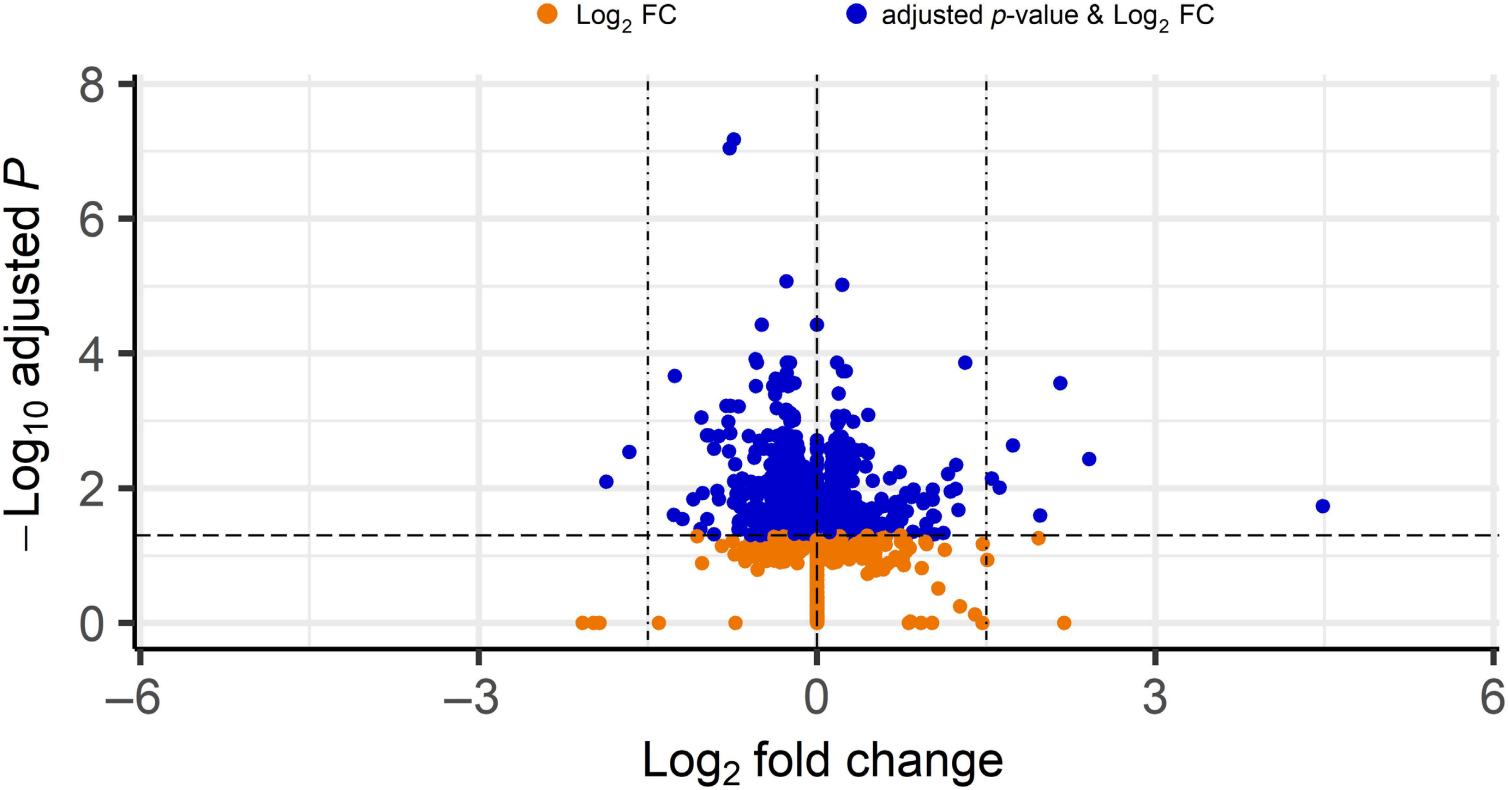
Volcano plot of results from the differential expression analysis with an adjusted *p*‐value cut‐off of .05 and log_2_ fold change cut‐off of 0. Each dot represents a gene. The horizontal dashed line is the adjusted *p*‐value cut‐off of .05, shown in negative log10 scale. The center vertical dashed line is the log_2_ fold change cut‐off of zero and the two vertical dot‐dash lines represent the cut‐off where the majority of differentially expressed genes fell (log_2_ FC = ±1.5). Only those genes that meet both the log_2_ fold change and adjusted *p*‐value cut‐offs are considered as significantly differentially expressed (blue dots). Log_2_ FC: significant by log_2_ fold change cut‐off only (orange dots), adjusted *p*‐value and log_2_ FC: significant by both adjusted *p*‐value and log_2_ fold change cut‐offs (blue dots).

**FIGURE 6:**
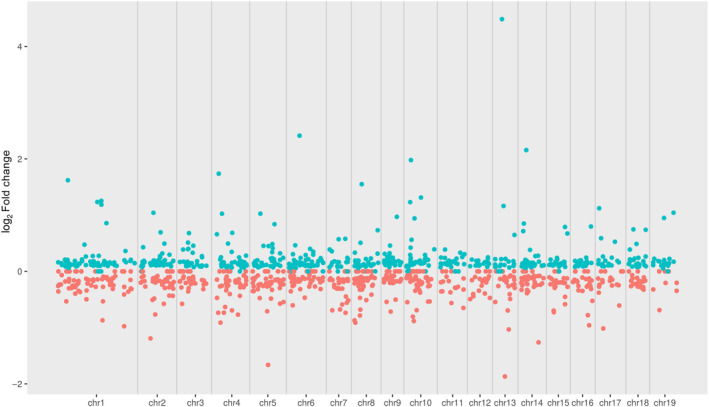
Distribution of differentially expressed genes across *Populus tremula* genome assembly v2.2. Dots represent differentially expressed genes, with blue dots being upregulated and red dots being downregulated.

Differential expression revealed 1243 differentially expressed genes, with 587 upregulated and 656 downregulated in the high PG genets relative to the low PG genets, out of a total of 30,249 genes with non‐zero total read counts. The volcano plot (Figure 5) shows that most of the significantly (FDR = 0.05) differentially expressed genes have log_2_ fold changes of less than 1.5. In other words, they are up‐ or down‐regulated ~3× (2^1.5^) or less in the high PG group as compared with the low PG group. Differentially expressed genes were spread across the genome (Figure 6).

We identified a large number of differentially expressed genes, so we used soft clustering methods to group them. We identified 13 gene clusters, of which seven clusters were significantly enriched for GO terms (Table 4, File S8). Most of the enriched clusters contained groups of genes that were associated with general biological processes such as photosynthesis or protein synthesis. Interestingly, clusters with growth‐related functions were downregulated in the high PG group as compared with the low PG group.

**TABLE 4:** Soft clustering analysis of differentially expressed gene results; genes included must display a membership value of 0.75 or higher (See File S9 for more details).

Cluster	Description	Number of genes	Expression pattern relative to the low PG group
C1^a^	DNA and RNA processes	67	Down
C2^a^	Photosynthesis	41	Down
C3	Enzymes related to sugar metabolism and other processes	53	Down
C4	Phenylpropanoid biosynthesis	69	Up
C5^a^	Protein synthesis and catabolism	89	Up
C6	Enzymes	62	Up
C7^a^	Protein synthesis and transport, enzymes	119	Down
C8^a^	Tetrapyrrole biosynthesis	34	Down
C9	Growth	58	Down
C10	Growth	52	Down
C11^a^	Secondary metabolism and regulation	128	Up
C12^a^	RNA and ribosome processes	94	Up
C13	Biosynthesis and regulation	44	Down

^a^ Displayed significant enrichment for GO annotations (See File S8 for more details).

Three clusters (C4, C11, C13) contained genes of clear interest because of their association with phenylpropanoid biosynthesis and regulation (Table 4), one (C11) of which was enriched for GO terms in secondary metabolism and regulation. Genes in two of those clusters (C4 and C11), containing 69 and 128 genes respectively, were upregulated in the high PG group. The third cluster (C13) with 44 genes exhibited genes that were downregulated in the high PG group. These three clusters contained several transcription factors (MYB, WRKY, NAC) and enzymes (caffeoyl shikimate esterase‐like, cinnamoyl‐CoA reductase 1‐like) associated with phenylpropanoid biosynthesis, most of which were upregulated in the high PG group.

## 4 DISCUSSION

Forest tree genomics research in the last decade has revealed that the genetic architecture of most ecologically important traits remains largely unexplained using traditional GWA methods (Lind et al., 2018). Moreover, what has been ascertained is that most ecologically important traits likely have a polygenic basis where many loci of small to moderate effect explain much of the phenotypic variance (Lind et al., 2018). While it is likely that there are multiple non‐mutually exclusive explanations behind this “missing heritability”, it is undeniable that a mismatch exists between the polygenic architectures of most ecologically important traits and the traditional methods currently used to characterize their genetic architectures (Josephs et al., 2017; Lind et al., 2018). Consequently, our understanding of what genes underly ecologically important traits remains poor. Studies such as this that incorporate modified GWA and complementary methods to characterize the genetic architecture of ecologically important traits will provide a more complete picture.

### 4.1 Most aspen growth and defense traits have a polygenic architecture

Our exploration of the underlying genetic architecture of important traits in aspen (*P. tremuloides*) reveals that many of them are likely polygenic. We found relatively few trait‐SNP associations across the 30 growth and defense traits analyzed, despite most of our traits displaying high broad‐sense heritabilities. Most of the significant SNPs had low effect sizes and low to moderate frequency alleles (File S7). Furthermore, 22 candidate genes identified across all traits, excluding sex, are spread across the genome (Figure 4) and many of the candidate genes have regulatory roles (e.g., transcription factors or enzymes) and are multi‐functional. Almost all of the 12 candidate genes associated with defense‐related phytochemistry and damage traits have regulatory roles in growth, defense, or response to stress. Often these regulatory genes were members of gene families that regulate multiple cellular and biological processes (e.g., Potra002739g19881, a nudix hydrolase 18). One gene (Potra004005g24127, F‐box/kelch‐repeat protein At1g55270) was also associated with both damage and phytochemistry traits. The multi‐functional nature of these candidate genes emphasizes how interconnected the gene regulatory networks are likely to be for quantitative traits.

Unlike single‐locus GWA, multilocus GWA is not subject to the Winner's Curse, resulting in inflated effect sizes for significant loci (Josephs et al., 2017). Multilocus GWA also often explains far more phenotypic variation in traits than single‐locus GWA (Josephs et al., 2017). Furthermore, they can provide a more nuanced view of the complex genetic architectures of quantitative traits, which can be used to adapt study design and analysis methods to better detect candidate genes underlying these traits moving forward. Our multilocus GWA results revealed that most of our traits exhibited a polygenic architecture, with generally low narrow‐sense heritability (h^2^) values and relatively high n_gamma values. For example, the genetic architecture of defense‐related phytochemistry traits showed that most of the variation explained by our marker set originates from loci with polygenic (i.e., infinitesimal) effects.

The one other study that used the multilocus GWA model, BSLMM, in a closely related *Populus* species found similar polygenic architecture of six comparable traits, including the main *Populus* defense phytochemicals. Bresadola et al. (2019) performed a multilocus GWA using RAD sequence data from a hybrid zone between closely related species to *P. tremuloides*: *P. tremula* and *P. alba*. Six traits were comparable between their study and ours, including the four salicinoid phenolic glycosides (salicin, salicortin, tremulacin, and tremuloidin), individual leaf area, and height. They found similar polygenic architectures for all six comparable traits (posterior distributions for our study (Figure S5) were compared to posterior distributions available in Bresadola et al. (2019) (Figure S8). Both Bresadola's and our study present a genetic architecture for these phytochemicals where most of the phenotypic variation is accounted for by many loci of small effects that are not likely to be detected by traditional GWA methods. The genes underlying the salicinoid phenolic glycoside biosynthesis pathway have been largely elusive (Fellenberg et al., 2020). Recent studies that have identified and validated candidate genes underlying salicinoid phenolic glycosides have used methods that can better account for a polygenic architecture (Fellenberg et al., 2020; Gordon et al., 2022). Taken together, our work and these studies underscore the importance of accounting for the polygenic nature of most ecologically important traits in characterizing their genetic architectures accurately.

### 4.2 Differentially expressed genes are associated with defense‐related phytochemistry

Transcriptomics is fast becoming a complementary method to traditional GWA to identify candidate genes in quantitative traits because of increasingly affordable sequencing costs and no need for extensive species‐specific genomic resources (Lind et al., 2018). Our study used differential expression analysis to explore expression patterns in low growth‐high defense and high growth‐low defense genotypes, identifying 197 upregulated and 44 downregulated genes of interest. Specifically, genes encoding enzymes from the phenylpropanoid biosynthesis pathway, upstream of the branches that produce salicinoid phenolic glycosides and condensed tannins, are present. Several transcription factors (e.g., WRKY, MYB, and NAC transcription factors) that are potentially involved in phytochemical defense and lignin formation were also differentially expressed between the low and high PG groups. Recent literature indicates potential co‐regulation of lignin and phytochemical defense via interconnected expression networks in plants (Xie et al., 2018; Zhang et al., 2018).

Other enzymes and transcription factors within our differentially expressed candidate gene list may, upon post‐GWA validation, prove to play a role in the still poorly understood salicinoid phenolic glycoside biosynthesis pathway. For example, (Fellenberg et al., 2020) used transcriptomics to identify candidate genes involved in the salicinoid biosynthesis pathway in *P. trichocarpa*. They identified a UDP‐glycosyltransferase gene and validated its essential role in the synthesis of major salicinoids through a CRISPER/Cas9‐engineered knockout experiment (Gordon et al., 2022). Three UDP‐glycosyltransferase genes were identified in our differential expression analysis that warrant further investigation.

Most of the differentially expressed genes identified have regulatory roles as transcription factors or enzymes that are often involved in more than one biological process. For example, one of the differentially expressed genes from the cluster enriched for genes involved in secondary metabolite regulation included a WRKY transcription factor 40 (Potra000926g07521) that was upregulated in the high PG group. This gene has a putative function in response to salicylic acid and regulation of biotic defense. As a group, WRKY transcription factors are often involved in response to biotic and abiotic stress (Jiang et al., 2014) and also in regulation of lignification (Wang et al., 2010). More recently, a WRKY transcription factor was shown to coregulate lignin biosynthesis and defense response in *Populus trichocarpa* (Zhang et al., 2018). These results emphasize how complex and intertwined the genetic architectures of ecologically important defensive traits likely are.

### 4.3 Relevance to “community genetics” and “genes‐to‐ecosystems” science

The framework proposed by Whitham et al. (2008) for identifying genes with community‐ and ecosystem‐level effects is similar to the original concept of using GWA analyses to identify genes underlying traits of interest in target organisms. Both had implicit assumptions that most traits of interest would be controlled by relatively few genes—assumptions bolstered by moderate to high predicted heritabilities for traits of interest. In reality, most variants associated with these highly heritable traits have exhibited small effect sizes indicating that the genetic architecture of most quantitative traits (which most ecologically important traits are) is likely polygenic (Bresadola et al., 2019; Chhetri et al., 2019; de la Torre et al., 2021, 2022; Lind et al., 2018). This may also be true for extended phenotypes, such as associated insect communities. In work concurrent with that reported here, Morrow (2022) found that several community metrics for herbivorous insects at the WisAsp common garden likely have undetected genetic associations—indicating that extended phenotypes themselves may exhibit polygenic architectures.

Our results provide gene‐level information about ecologically important traits that have been connected to associated communities and ecosystems. Genotype‐mediated growth‐defense trade‐offs are well established in aspen (Cole et al., 2021; Cope et al., 2019; Osier & Lindroth, 2006) and have been shown to differentially affect population genetic composition in contrasting environments (Cope et al., 2021). Moreover, divergent growth and defense phenotypes in aspen influence the community metrics of associated insect assemblages (Barker et al., 2019; Morrow, 2022). For example, a gibberellin 3‐beta‐dioxygenase gene (Potra003968g23830) associated with salicinoid phenolic glycosides in our GWA analyses has also been linked to the regulation of growth and defense responses in *Solanum* and *Nicotiana* spp. (Bhattacharya et al., 2012). We also identified 197 genes upregulated in genets with high foliar concentrations of salicinoid phenolic glycosides, including several genes involved in the potential regulation of phytochemical defense and lignin biosynthesis.

### 4.4 On the omnigenic model of polygenic architecture

A current expectation of genetic architecture is that the genes underlying trait variation would all cluster in key pathways related to the trait. Many polymorphisms are often found in non‐coding and regulatory regions of the genome and the omnigenic model (Boyle et al., 2017) suggests that a vast number of highly connected regulatory genes may explain most of the heritability of a trait through their modulation of the expression of a relatively smaller number of core genes. In other words, if gene regulatory networks are highly interconnected, then even a peripheral gene is likely to have a non‐zero effect on the expression of the trait. In this case, the expression of these peripheral genes will vastly outnumber the core genes and small effects of peripheral genes will quickly add up to account for far more trait variation than the core genes alone. As an example, Boyle et al. (2017) demonstrate that ~100,000 causal variants affect human height, and most are located in gene regulatory regions.

Many forest tree GWA results exhibit a similar pattern, finding mostly regulatory genes that often have vague known biological connections to the trait of interest (Barker et al., 2019; Bresadola et al., 2019; Chhetri et al., 2019; de la Torre et al., 2019, 2021, 2022; Fahrenkrog et al., 2017; Hallingbäck et al., 2019; Mähler et al., 2017, 2020; McKown et al., 2018). In particular, a recent co‐expression network analysis of budbreak in a closely related species, *P. tremula*, found a negative relationship between eQTL effect size and network connectivity, and that genes with low connectivity were enriched for eQTLs (Mähler et al., 2017). That work suggests that selection on peripheral genes was more relaxed than on core genes and provides a way to allow potentially adaptive mutations while buffering the core genes from potentially deleterious mutations. If this is the case for other quantitative tree traits, then most phenotypic variation in polygenic traits will be explained by peripheral genes that will be difficult to connect to traits of interest without a deeper understanding of the gene networks controlling them. In fact, one study has recently used a system genetics approach (e.g., co‐expression, eQTL analysis, gene regulatory network inference; Fagny & Austerlitz, 2021) to show that the genetic architecture of leaf shape variation in the closely related *P. tremula* follows the omnigenic model (Mähler et al., 2020).

Our GWA results share many of the characteristics associated with the omnigenic model of polygenic architecture. Most of our candidate genes have regulatory roles, exhibit low effect sizes, and are spread across the genome. Furthermore, multilocus GWA revealed most traits exhibited a polygenic architecture with many small effect loci explaining most of the phenotypic variation. While our results are likely impacted by our sample size and lack of whole‐genome coverage as detailed below, they are consistent with other studies in similar species that have more complete genome coverage (Chhetri et al., 2019; Escamez et al., 2021; McKown et al., 2018; PK Ingvarsson unpublished data). Still, it must be noted that other factors such as sample size (Lind et al., 2018), effect size biases (Josephs et al., 2017), and accounting for effects of other non‐coding variants (e.g., structural variants; Holliday et al., 2017) need to be considered as the field progresses.

### 4.5 Study limitations

Like many forest tree GWA studies, our sampling and genomic resources have limitations. Our sample size (*N* = 455) was sufficiently large for most GWA studies. However, recent studies in forest trees have shown that alleles for large‐effect loci are likely to be rare, requiring sample sizes in the thousands to detect them (Josephs et al., 2017; Mähler et al., 2017). Thus, we likely are unable to detect rare variants of large effects. We sequenced the exome, so our marker dataset did not cover the entire genome. Additionally, some genes could not be sequenced if the probe did not map to a unique genomic location, an issue common in species, like *Populus* (Berlin et al., 2010), with whole‐genome duplications in their evolutionary history. Thus, we are likely missing some genes as well as the non‐coding regions. We believe, however, that our incomplete genomic coverage does not adversely affect our main interpretations. Several studies in *Populus* species using whole‐genome sequence data have similarly found fewer than expected associations for highly heritable traits when using traditional GWA methods (Chhetri et al., 2019; Escamez et al., 2021; McKown et al., 2018; PK Ingvarsson unpublished data). In short, finding associations that explain a substantial amount of phenotypic variation seems to be the exception, rather than the rule, for most quantitative traits in forest trees.

### 4.6 Conclusions

Despite the challenges presented over the last couple of decades in applying genomics to understanding fundamental molecular and ecological processes, we have come a long way in understanding the complexity of the genetic architecture underlying ecologically important traits. Traditional single‐locus GWA studies in forest trees have had limited success in uncovering the genomic underpinnings of many ecologically important, quantitative traits. Studies of both plants and humans reveal that the genetic architecture of most quantitative traits is likely polygenic and that regulatory genes in the periphery of gene networks may play a large role in controlling trait variation (Fagny & Austerlitz, 2021; Visscher et al., 2017). Our study is one of very few to incorporate several years of phenotypic data across many ecologically important traits from a large common garden for a species with little to no population structure. Additionally, our study is one of the first to employ multilocus GWA for a forest tree species (also see Bresadola et al., 2019; de la Torre et al., 2021). This work adds to a growing body of evidence that many ecologically important traits in forest trees are polygenically controlled, with many genes of small effect underlying phenotypic variation.

## AUTHOR CONTRIBUTIONS

**Jennifer F. L. Riehl:** Conceptualization (equal); data curation (equal); formal analysis (equal); funding acquisition (equal); investigation (equal); methodology (lead); project administration (lead); validation (equal); visualization (lead); writing – original draft (lead); writing – review and editing (lead). **Christopher T. Cole:** Data curation (equal); formal analysis (equal); investigation (equal); methodology (supporting); project administration (equal); validation (equal); visualization (supporting); writing – review and editing (supporting). **Clay J. Morrow:** Data curation (equal); formal analysis (equal); investigation (equal); methodology (supporting); project administration (equal); validation (supporting); visualization (supporting); writing – review and editing (supporting). **Hilary L. Barker:** Data curation (supporting); formal analysis (supporting); investigation (supporting); methodology (equal); writing – review and editing (supporting). **Carolina Bernhardsson:** Data curation (supporting); formal analysis (equal); investigation (supporting); methodology (equal); validation (equal); writing – review and editing (supporting). **Kennedy Rubert‐Nason:** Data curation (supporting); investigation (supporting); project administration (supporting); validation (supporting); writing – review and editing (supporting). **Pär K. Ingvarsson:** Conceptualization (equal); data curation (supporting); formal analysis (supporting); funding acquisition (equal); investigation (supporting); methodology (supporting); project administration (supporting); supervision (equal); writing – review and editing (equal). **Richard L. Lindroth:** Conceptualization (equal); data curation (supporting); formal analysis (supporting); funding acquisition (equal); investigation (supporting); methodology (supporting); project administration (supporting); supervision (equal); writing – review and editing (equal).

## BENEFIT‐SHARING STATEMENT

Benefits from this research accrue from the sharing of our data and results on public databases as described above.

## Supporting information


File S1.

File S2.

File S3.

File S4.

File S5.

File S6.

File S7.

File S8.

Figure S1.

Figure S2.

Figure S3.

Figure S4.

Figure S5.

**Tables S1.**–**S2.**

## Data Availability

WisAsp variant plink and GEMMA format files, phenotype data, and DESeq2 format files: Dryad DOI: 10.5061/dryad.9zw3r22jr. Raw DNA sequence data for all samples included in the genome‐wide association analyses are available through the European Nucleotide Archive under accession number PRJEB30919. Raw RNA sequence data for all samples included in the differential expression analysis are available through the National Center for Biotechnology Information's Sequence Read Archive under accession number PRJNA851830.
