# Supplementary material for: Genomic and transcriptomic analyses reveal polygenic architecture for ecologically important traits in aspen (Populus tremuloides Michx.)
Source: Ecol Evol. 2023 Sep 28;13(10):e10541. doi: 10.1002/ece3.10541 (PMC10534199; doi:10.1002/ece3.10541)
Supplement: Supplementary file 3 — File S3. [file ECE3-13-e10541-s010.docx]

**Supplemental File S3**

Supplemental File S3 provides detailed information on the RNA-seq sequencing data filtering pipeline and differential expression analysis. All referenced input files can be accessed on Dryad (DOI: <https://doi.org/10.5061/dryad.9zw3r22jr>) unless otherwise noted.

***Steps 1-6: RNA-seq sequencing data filtering pipeline (completed using Linux command line)***

*Step One: Quality Assessment using FASTQC and multiqc*

*Script to run*

**fastqc -o ~/output/location -t 16 ~/input/location/*.fastq.gz**

**multiqc .**

*Explanation of script*

**fastqc** call program

**-o ~/output/location** specify the location to which the output files should be stored

**-t 16** **~/input/location/*.fastq.gz** specify the location of the input files and how many to process simultaneously

**multiqc .** calls program and parses summary statistics from results and log files generated by other bioinformatics tools using the current working directory

*Step Two: rRNA removal via SortMeRna*

*Script to run*

**time find ../20190214_mRNASeq_PE150/ -name "*.fastq.gz" | sort | while read READ_FW; do read READ_RV; bash /home/jfriehl/bin/runSortMeRNA.sh $READ_FW $READ_RV; done**

*Explanation of script*

**time** will determine how long a given command will take (used to assess program efficiency)

**find ../20190214_mRNASeq_PE150/ -name "*.fastq.gz"** locate input files

**|** take output from previous command and use it in following command

**sort** sort the input files in a particular order

**while read READ_FW; do read READ_RV;** passes filenames as arguments in a given script

**bash /home/jfriehl/bin/runSortMeRNA.sh $READ_FW $READ_RV;** calls script to run through the steps of using the SortMeRna program to filter out ribosomal RNA sequences from the filenames specified in the previous command

**done** closes the while loop

*Step Three: Quality Assessment using FASTQCand mutliqc*

*Script to run*

**fastqc -o ~/output/location -t 16 ~/input/location/*.fastq.gz**

**multiqc .**

*Explanation of script*

**fastqc** call program

**-o ~/output/location** specify the location to which the output files should be stored

**-t 16** **~/input/location/*.fastq.gz** specify the location of the input files and how many to process simultaneously

**multiqc .** calls program and parses summary statistics from results and log files generated by other bioinformatics tools using the current working directory

*Step Four: Quality-based trimming and adapter removal via Trimmomatic*

*Script to run*

**find ../Sortmerna/ -name "*sortmerna_[12].fastq.gz" | sort | while read FW_READ; do read RV_READ; bash /home/jfriehl/bin/runTrimmomatic.sh $READ_FW $READ_RV; done**

*Explanation of script*

**find ../Sortmerna/ -name "*sortmerna_[12].fastq.gz"** locate input files

**|** take output from previous command and use it in following command

**sort** sort the input files in a particular order

**bash /home/jfriehl/bin/runTrimmomatic.sh $READ_FW $READ_RV;** calls script to run through the steps of using the Trimmomatic program to filter out low quality and adaptor sequences from the filenames specified in the previous command

**done** closes the while loop

*Step Five: Quality Assessment using FASTQC and multiqc*

*Script to run*

**fastqc -o ~/output/location -t 16 ~/input/location/*.fastq.gz**

**multiqc .**

*Explanation of script*

**fastqc** call program

**-o ~/output/location** specify the location to which the output files should be stored

**-t 16** **~/input/location/*.fastq.gz** specify the location of the input files and how many to process simultaneously

**multiqc .** calls program and parses summary statistics from results and log files generated by other bioinformatics tools using the current working directory

*Step Six: Quasi-alignment to the P. tremula genome assembly v2.2 via SALMON*

*6a)* *First, build a Salmon index from a reference transcriptome.*

*Script to run*

**bash /home/jfriehl/Documents/Expression_Projects/Scripts/generateDecoyTranscriptome.sh -a /home/jfriehl/Documents/Expression_Projects/Reference_materials/Reference_Fasta/Potra02/Potra02_genes.gff -g /home/jfriehl/Documents/Expression_Projects/Reference_materials/Reference_Fasta/Potra02/Potra02_genome.fasta -t /home/jfriehl/Documents/Expression_Projects/Reference_materials/Reference_Fasta/Potra02/Potra02_transcripts.fasta -o /home/jfriehl/Documents/Expression_Projects/Reference_materials/Reference_Fasta/Salmon/Potra_decoys**

**salmon index -t Potra02_transcripts.fasta -i Potra02_transcripts_index -decoys Potra_decoys.txt -k 31**

*Explanation of script*

**bash /home/jfriehl/Documents/Expression_Projects/Scripts/generateDecoyTranscriptome.sh** calls script to be run using awk, bedtools and mashmap

**-a ../Potra02_genes.gff** specify gff/gtf file

**-g ../Potra02_genome.fasta** specify genome assembly fasta file

**-t ../Potra02_transcripts.fasta** specify transcripts fasta file

**-o ../Potra_decoys** specify location and name of output file

**salmon** call program

**index -t Potra02_transcripts.fasta -i Potra02_transcripts_index -decoys Potra_decoys.txt -k 31**  build the mapping-based index, with a minimum acceptable length or k size for a valid match of 31 (standard that works well for reads 75bp or longer)

**NOTE:** A decoy-aware transcriptome needs to be built to account for reads that may map to an annotated transcript when they really came from a transcript that isn’t annotated.

**NOTE:** Salmon by default removes duplicate transcripts (see here for discussion <https://github.com/COMBINE-lab/salmon/issues/214>). We used the default method, since one can recover the abundances for the duplicate transcripts post hoc if necessary. For our specific dataset, SALMON removed 171 transcripts from *P. tremula* v2.2 transcriptome.

*6b) Quantify paired-end reads against the Salmon index*

*Script to run*

**find ../Trimmomatic/ -name "*sortmerna-trimmomatic_[12].fastq.gz" | sort | while read FW_READ; do read RV_READ; bash /home/jfriehl/bin/ Salmon_quant_Potra.sh.sh $READ_FW $READ_RV; done**

*Explanation of script*

**find ../Sortmerna/ -name "*sortmerna_[12].fastq.gz"** locate input files

**|** take output from previous command and use it in following command

**sort** sort the input files in a particular order

**bash /home/jfriehl/bin/ Salmon_quant_Potra.sh.sh $READ_FW $READ_RV;** calls script to be run using salmon to quantify RNA-seq data via Salmon

**done** closes while loop

**NOTE:** All shell scripts referenced above are available upon request.

***Step Seven: Differential expression analysis (completed using R/RStudio and various Bioconductor packages including DEseq2)***

**NOTE:** The following tutorial was used to guide this process: <https://www.bioconductor.org/packages/devel/workflows/vignettes/rnaseqGene/inst/doc/rnaseqGene.html>

*Step 7a) Importing count data via* tximport

This step prepares the metadata and summarizes RNA-seq experiment as a count matrix.

*Step 7b) Biological QC via DEseq2, pheatmap, and PCAtools*

This step assesses whether observed effects may have biological causes (e.g., are conditions grouped together or sufficiently separated, are there any obvious confounding factors such as sequencing flow cell or lane, etc.). This was done by visualizing the data clustering patterns via heatmaps and PCA analyses using normalized counts to ensure there is a roughly equal contribution from all genes.

For this study, ramets of each genet were pooled prior to RNA sequencing. We have two examples of samples that turned out to be ramets of one another that were sent separately for sequencing. These samples exhibited highly similar expression profiles compared to genets that were not clones of one another. Thus, the differences among ramet expression profiles are small compared to differences among genet expression profiles and the pooling of ramets did not lead to a loss of information. At the same time, separation of the high and low PG genets was not clear in a heatmap assessment of the first 50 rows of the count matrix, which was also confirmed by the PCA analysis showing distinct groupings of the high and low PG groups with overlap between the two groups. Given the pseudo-condition set-up of our sample collection, we expected the data to be a bit noisy.

*Step 7c) Differential expression analysis via DESeq2*

First, a negative binomial generalized linear model is fitted to perform the differential expression. The estimates of dispersion and logarithmic fold changes incorporate data-driven prior distributions.

From the DESeq2 Manual:

*Kij*∼NB(*μij*,*αi*)

where counts *Kij* for gene i, sample j are modeled using a negative binomial distribution with fitted mean *μij* and a gene-specific dispersion parameter *αi*

DESeq2 automatically assesses the data for outliers and filter for genes with low expression or no expression levels across all or most samples. To assess outliers, a diagnostic test, Cook’s distance, is applied. Cook’s distance is a measure of how much a single is influencing the fitted coefficients for a gene, with a large value of Cook’s distance intended to indicate an outlier count. Our samples all have small values for Cook’s distance, so there are no outlier counts due to errant samples.

In terms of filtering out genes with low or no expression, we can look at some basic statistics (i.e., baseMean or normalized counts and log2 fold change or effect sizes) post filtering to see if any further filtering should be done. The lowest our log2 fold change value is around 0.06; 1,668 of the 2,087 differentially expressed genes have a log2 fold change < 0.5 and > -0.5, but all genes with a baseMean < 5 have a log2 fold change > 1 and for those genes with log2 fold change between 0.5 and -0.5, the minimum baseMean value is ~11. Thus the default filtering is adequately filtering our dataset for genes with low (i.e., low baseMean and log2 fold change) or no expression.

We also applied the apeglm shrinkage method from the package apeglm (now the default for DESeq2, Zhu, Ibrahim, and Love 2018) to reduce experimental noise and preserve highly differentially expressed genes. Shrinkage of effect sizes (log2 fold change estimates) reduces false positives in differential expression analysis by accounting for genes with low dispersion estimates by shrinking them towards the curve and generating more accurate log2 fold change estimates for fitting the model and differential expression testing.

We have chosen to apply independent hypothesis filtering to adjust our p-values and account for multiple testing. Independent hypothesis weighting (IHW) is a multiple testing procedure that allows the inclusion of an informative and independent covariate for each test. Since baseMean (i.e., the mean of normalized counts) and p-values generated from differential expression analysis are approximately independent under the null hypothesis (DESeq2 paper), we can use baseMean as the covariate. Data-driven hypothesis weighting has been shown to increase detection power in multiple testing analyses like differential expression (Ignatiadis et al, 2016).

For the differential expression we applied an IHW cut-off of 0.05 and a log2 fold change cut-off of zero (i.e., keep results with a log2 fold change value of either more than (upregulated) or less than zero (downregulated). We followed Schurch et al (2016), which recommends that a log2 fold cut-off of zero is acceptable for experiments with sample sizes of more than 12 per condition.

**NOTE:** The differential expression R script is available upon request.

**References**

Andrews, S. (2010) FastQC: a quality control tool for high throughput sequence data. <https://www.bioinformatics.babraham.ac.uk/projects/fastqc/>

Bolger, A. M., Lohse, M., & Usadel, B. (2014) Trimmomatic: a flexible trimmer for Illumina sequence data. *Bioinformatics*, 30: 2114-2120. DOI: <https://doi.org/10.1093/bioinformatics/btu170>

Ewels, P., Magnusson, M., Sverker, L., Käller, M. (2016) MultiQC: summarize analysis results for multiple tools and samples in a single report. Bioinformatics, 32:3047–3048. DOI: <https://doi.org/10.1093/bioinformatics/btw354>

Ignatiadis, N., Klaus, B., Zaugg, J.B., Huber, W. (2016) Data-driven hypothesis weighting increases detection power in genome-scale multiple testing. *Nature Methods*, 13:7. DOI: [10.1038/nmeth.3885](https://doi.org/10.1038%2Fnmeth.3885)

Kopylova, E., Noé, L., Touzet, H. (2012) SortMeRNA: Fast and accurate filtering of ribosomal RNAs in metatranscriptomic data. *Bioinformatics* 28:3211-3217. DOI: <https://doi.org/10.1093/bioinformatics/bts611>

Love, M.I., Huber, W. & Anders, S. (2014) Moderated estimation of fold change and dispersion for RNA-seq data with DESeq2. *Genome Biol* 15: 550. DOI: <https://doi.org/10.1186/s13059-014-0550-8>

Patro, R., Duggal, G., Love, M., Irizarry, R.A., Kingsford, C. (2017) Salmon provides fast and bias-aware quantification of transcript expression. *Nat Methods* 14:417–419. DOI: <https://doi.org/10.1038/nmeth.4197>

Schurch, N. J., Schofield, P., Gierliński, M., Cole, C., Sherstnev, A., Singh, V., Wrobel, N., Gharbi, K., Simpson, G. G., Owen-Hughes, T., Blaxter, M., & Barton, G. J. (2016). How many biological replicates are needed in an RNA-seq experiment and which differential expression tool should you use?. *RNA*, 22: 839–851. DOI: <https://doi.org/10.1261/rna.053959.115>

Zhu, A., Ibrahim, J. G., & Love, M. I. (2019). Heavy-tailed prior distributions for sequence count data: removing the noise and preserving large differences. *Bioinformatics* 35:2084–2092. DOI: <https://doi.org/10.1093/bioinformatics/bty895>
