# Supplementary material for: Genomic and transcriptomic analyses reveal polygenic architecture for ecologically important traits in aspen (Populus tremuloides Michx.)
Source: Ecol Evol. 2023 Sep 28;13(10):e10541. doi: 10.1002/ece3.10541 (PMC10534199; doi:10.1002/ece3.10541)
Supplement: Supplementary file 4 — File S4. [file ECE3-13-e10541-s006.docx]

**Supplemental File S4**

Supplemental File S4 provides the basic scripts for the multilocus (BSLMM) and single-locus (univariate and multi-trait) genome-wide association analyses using a Linux-based platform and command line. All referenced input files can be accessed on Dryad (DOI: <https://doi.org/10.5061/dryad.9zw3r22jr>) unless otherwise noted.

***Single-locus genome-wide association analyses using Plink 1.9***

*Univariate GWA*

*Script to run*

**plink --bfile WisAsp_MAF005 --assoc --no-sex --pheno WisAsp_Tree_Trait_BLUPs.txt --all-pheno --mperm 5000 --seed 105 --qt-means --adjust --out plink_MAF005_univariate_sibs**

*Explanation of script*

**plink** call program

**--bfile WisAsp_MAF005** tell program what file type to use for the genomic data; X is either 05 or 005 for 5% and 0.5% MAF filters respectively

**--assoc** performs simple linear regression using the Wald statistic to generate p-values

**--no-sex** indicates there is no sex field

**-- WisAsp_Tree_Trait_BLUPs.txt** tells program to use this file for phenotype information in place of a ped file (i.e., in case of using .fam files)

**--all-pheno** perform GWA for all traits in the file

**--mperm 5000** max(T) permutation; doesn’t drop SNPs like the adaptive method; less stringent than Bonferroni correction because it keeps correlational structure of SNPs (tests not independent); default is label swapping

**--seed 105** to allow reproducible results because it means the random numbers generated will be the same every time

**--qt-means** provides an additional file with a list of means and standard deviations stratified by genotype

**--adjust** adjusted significance values

**--out plink_MAF005_univariate_sibs** specify the name of the output file

*Multi-trait GWA*

*Script to run*

**plink.multivariate --noweb --file WisAsp_MAF005 --allow-no-sex --mqfam**

**-- WisAsp_Tree_Trait_BLUPs.phen --pheno-number C1,C2,C3,C4 --out plink_MAF005_multivariate_sibs_ MultivariateAbbreviation**

*Explanation of script*

**plink.multivariate** call program

**--noweb** skip connecting to the web version of plink

**--file WisAsp_MAF005** tell program what file to use for the genomic data

**--allow-no-sex** allow analysis to proceed without a sex field

**--mqfam** perform multivariate GWA (see here for more information: <https://genepi.qimr.edu.au/staff/manuelF/multivariate/main.html>)

**--mult-pheno WisAsp_Tree_Trait_BLUPs.phen** tells program to use this file for phenotype information in place of a ped file (i.e., in case of using .fam files)

**--pheno-number C1,C2,C3,C4** tells the program which columns numbers from the phenotype data file to use in the multi-trait GWA; should be written as integers with commas in between; column numbers associated with each trait can be found in Dryad_Lind-Riehl_etal_MolEcol_2023_metadata.xlsx under the tab “Tree trait file”

**--out plink_MAF005_multivariate_sibs_MultivariateAbbreviation** specify the name of the output file; MultivariateAbbreviation comes from the assigned list found in “Supplemental_File_S5.xlsx” under the tab “Multi-trait Trait Legend”

*Case/Control Univariate GWA*

NOTE: used this test for sex as it is more appropriate than quantitative trait association analysis; this option in plink uses Fisher’s Exact test to calculate p-values

*Script to run*

**plink --bfile WisAsp_MAF005 --assoc fisher --no-sex --pheno WisAsp_Tree_Trait_BLUPs.txt --all-pheno --mperm 5000 --seed 105 --out plink_MAF005_univariate_sibs_SEX.B_Fisher**

*Explanation of script*

**plink** call program

**--bfile WisAsp_MAF005** tell program what file type to use for the genomic data

**--assoc fisher** performs simple linear regression using Fisher’s exact test to generate p-values

**--no-sex** indicates there is no sex field

**--pheno WisAsp_Tree_Trait_BLUPs.txt** tells program to use this file for phenotype information in place of a ped file (i.e., in case of using .fam files)

**--all-pheno** perform GWA for all traits in the file

**--mperm 5000** max(T) permutation; doesn’t drop SNPs like the adaptive method; less stringent than Bonferroni correction because it keeps correlational structure of SNPs (tests not independent); default is label swapping

**--seed 105** to allow reproducible results because it means the random numbers generated will be the same every time

**--out plink_MAF005_univariate_sibs_SEX.B_Fisher** specify the name of the output file

***Multilocus genome-wide association analyses using GEMMA’s BSLMM model***

*Multi-locus GWA run on the CHTC (https://chtc.cs.wisc.edu/)*

JF Riehl created the following two scripts to run the multilocus GWA using GEMMA’s BSLMM model:

0.5% MAF cutoff

gemma_bslmm_MAF005.sub (available upon request) and gemma_bslmm_MAF005.sh

A final file called list.txt (available upon request) needs to be made that lists in one column a series of numbers equal to the number of traits you are planning to run through GEMMA’s BSLMM model

Once logged into the CHTC, run the following command to start the runs:

*[riehl2@submit3 ~]$* **condor_submit gemma_bslmm_MAF005.sub**

Output files will be large and need to be compressed before transferring from CHTC to the chosen work computer:

*[riehl2@submit3 ~]$* **tar cvfj BSLMM_MAF005_Run_1.tar.bz2 /home/riehl2/0/**

Follow the directions from the following help page to transfer files to and from the CHTC server: <http://chtc.cs.wisc.edu/connecting.shtml#transfer-scp>

**NOTE:** All referenced scripts are available upon request.

**References**

Purcell, S., Neale, B., Todd-Brown, K., Thomas, L., Ferreira, M.A.R., Bender, D., Maller, J., Sklar, P., de Bakker, P.I.W., Daly, M.J., Sham, P.C. (2007) PLINK: a toolset for whole-genome association and population-based linkage analysis. Am J Hum Genet, 81: 559-575. <https://www.cog-genomics.org/plink/1.9/>

Riehl, J. F. L., Cole, C. T., Morrow, C. J., Barker, H. L., Bernhardsson, C., Rubert‐Nason, K. F., Ingvarsson, P. K., & Lindroth, R. L. (2022). Data from: Genomic and transcriptomic analyses reveal polygenic architecture for ecologically-important functional traits in aspen (*Populus tremuloides* Michx.). Dryad Digital Repository, https://doi.org/10.5061/dryad.9zw3r22jr

Zhou, X., Carbonetto, P., & Stephens, M. (2013). Polygenic modeling with Bayesian sparse linear mixed models. *PLoS Genetics*, *9*(2). <https://doi.org/10.1371/journal.pgen.1003264>
