# Supplementary figures and images for: Genomic and transcriptomic analyses reveal polygenic architecture for ecologically important traits in aspen (Populus tremuloides Michx.)
Source: Ecol Evol. 2023 Sep 28;13(10):e10541. doi: 10.1002/ece3.10541 (PMC10534199; doi:10.1002/ece3.10541)

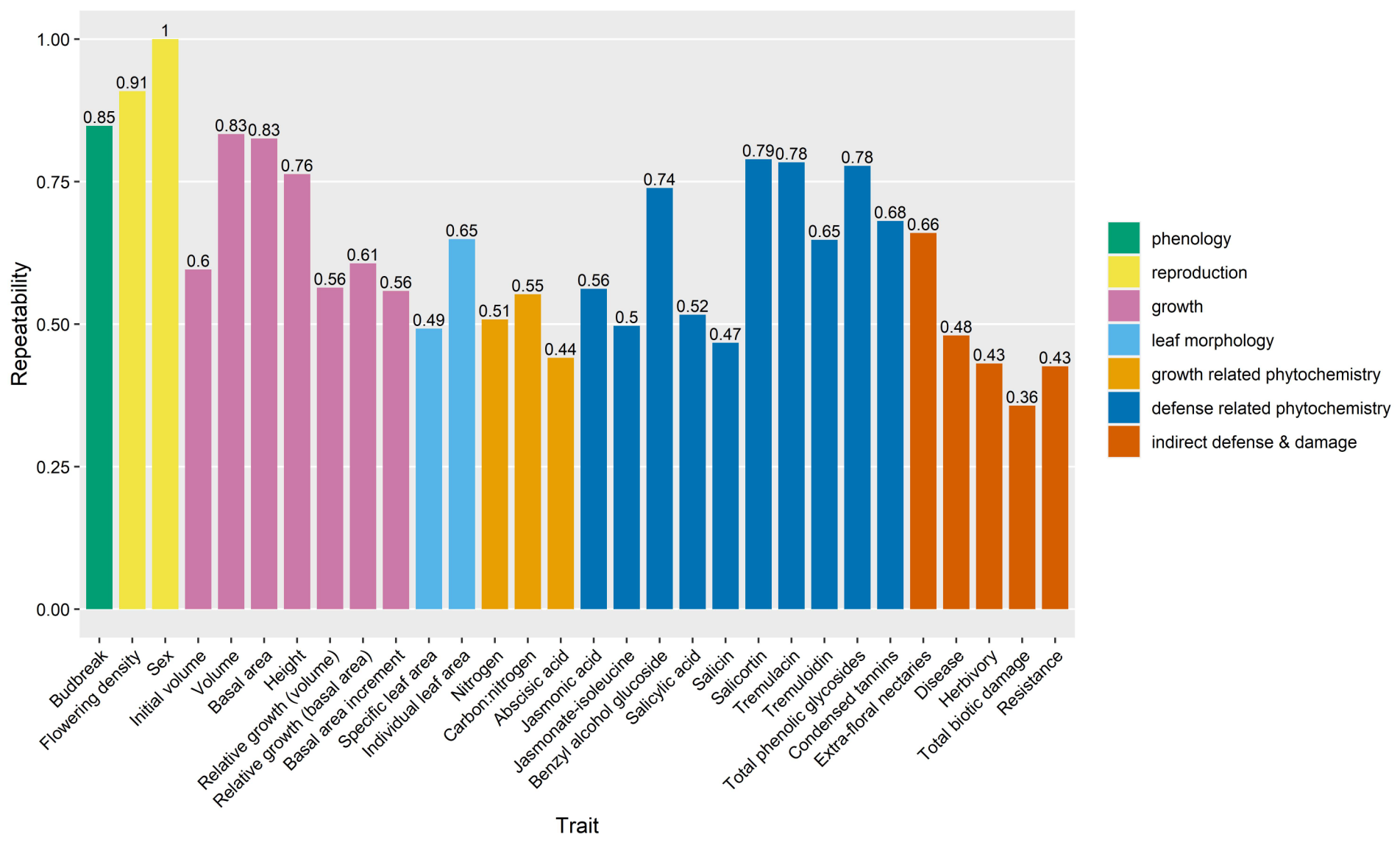

Supplement: Supplementary file 9 — Figure S1. [file ECE3-13-e10541-s008.pdf]

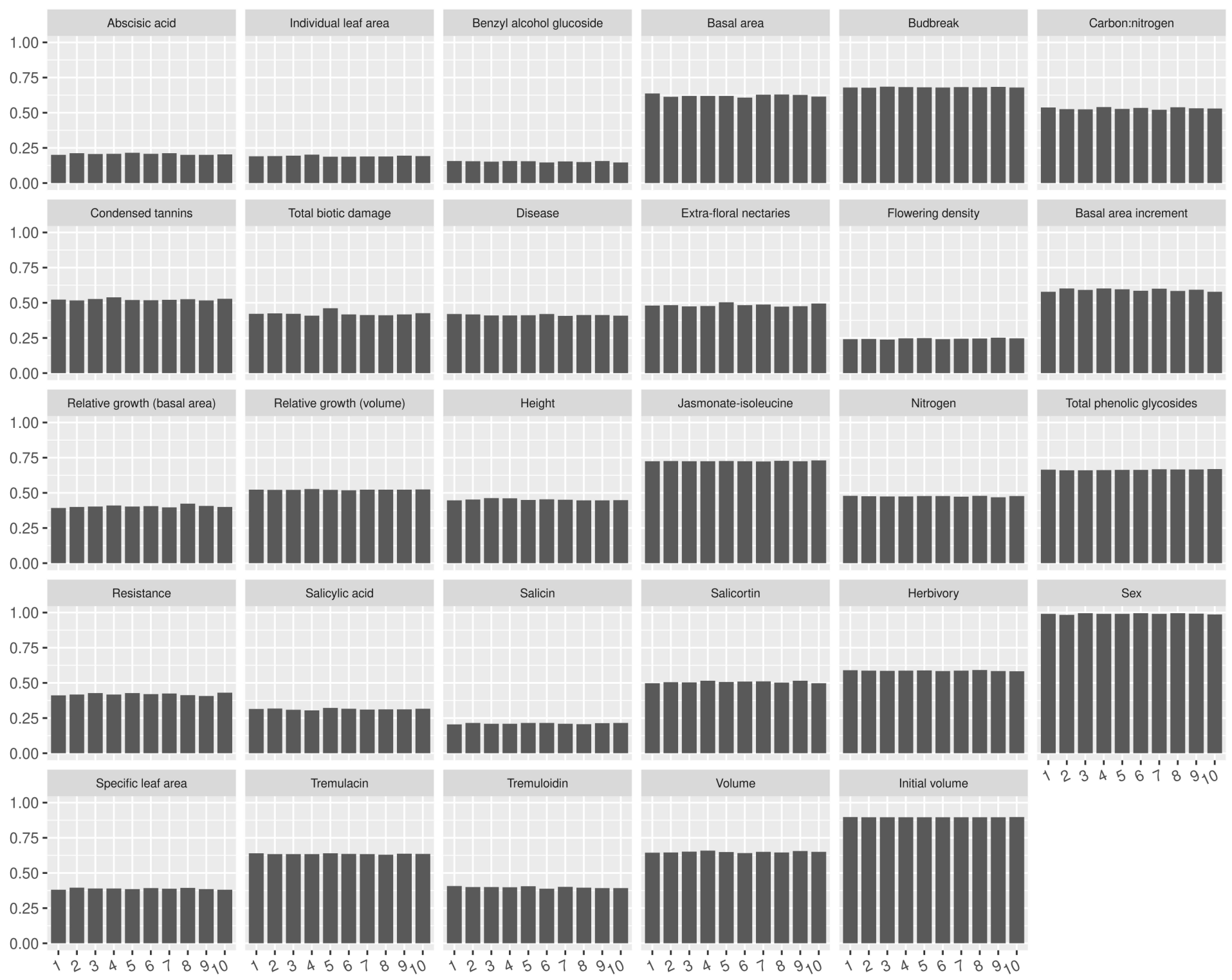

Supplement: Supplementary file 10 — Figure S2. [file ECE3-13-e10541-s005.pdf]

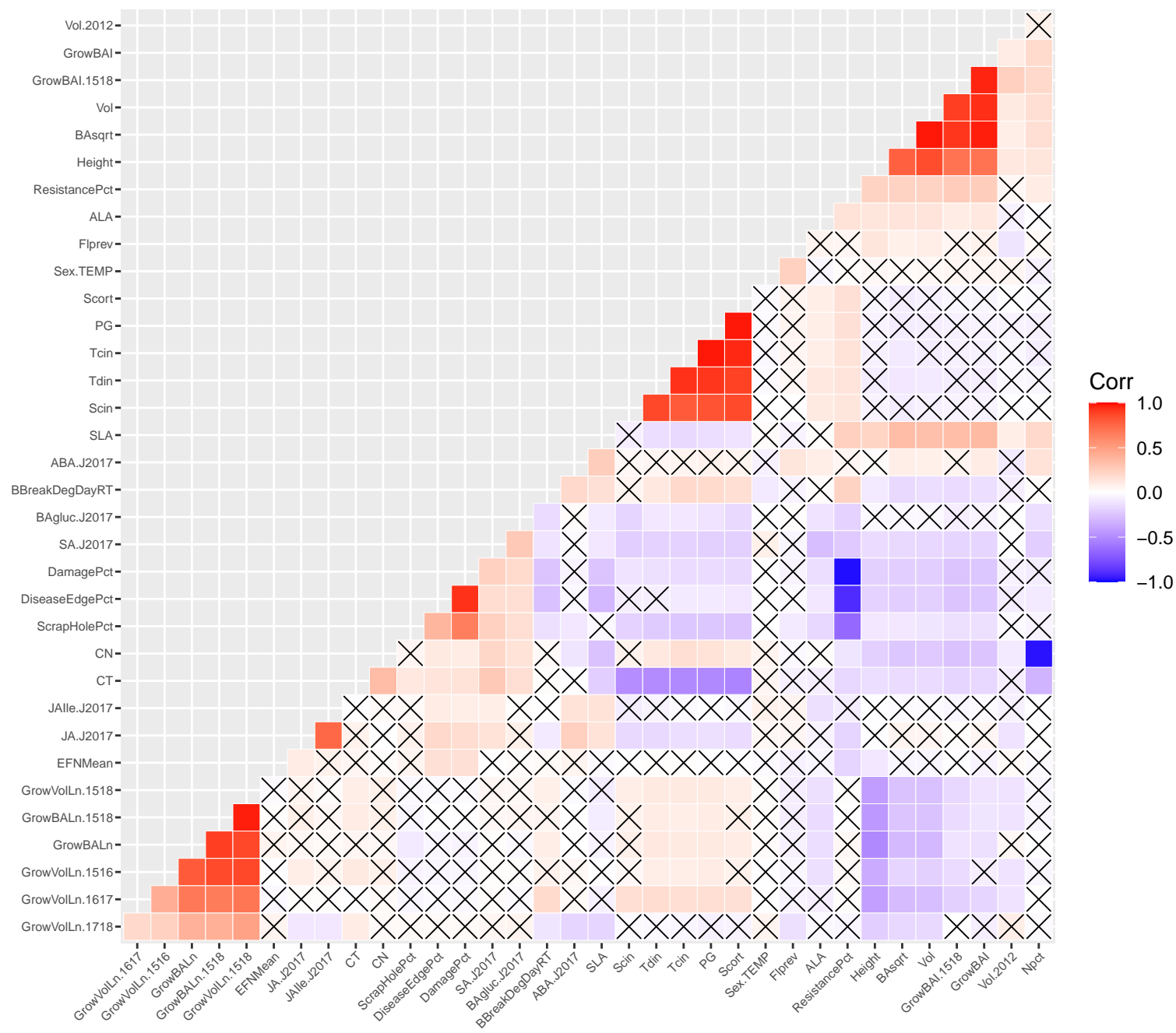

Supplement: Supplementary file 11 — Figure S3. [file ECE3-13-e10541-s002.pdf]

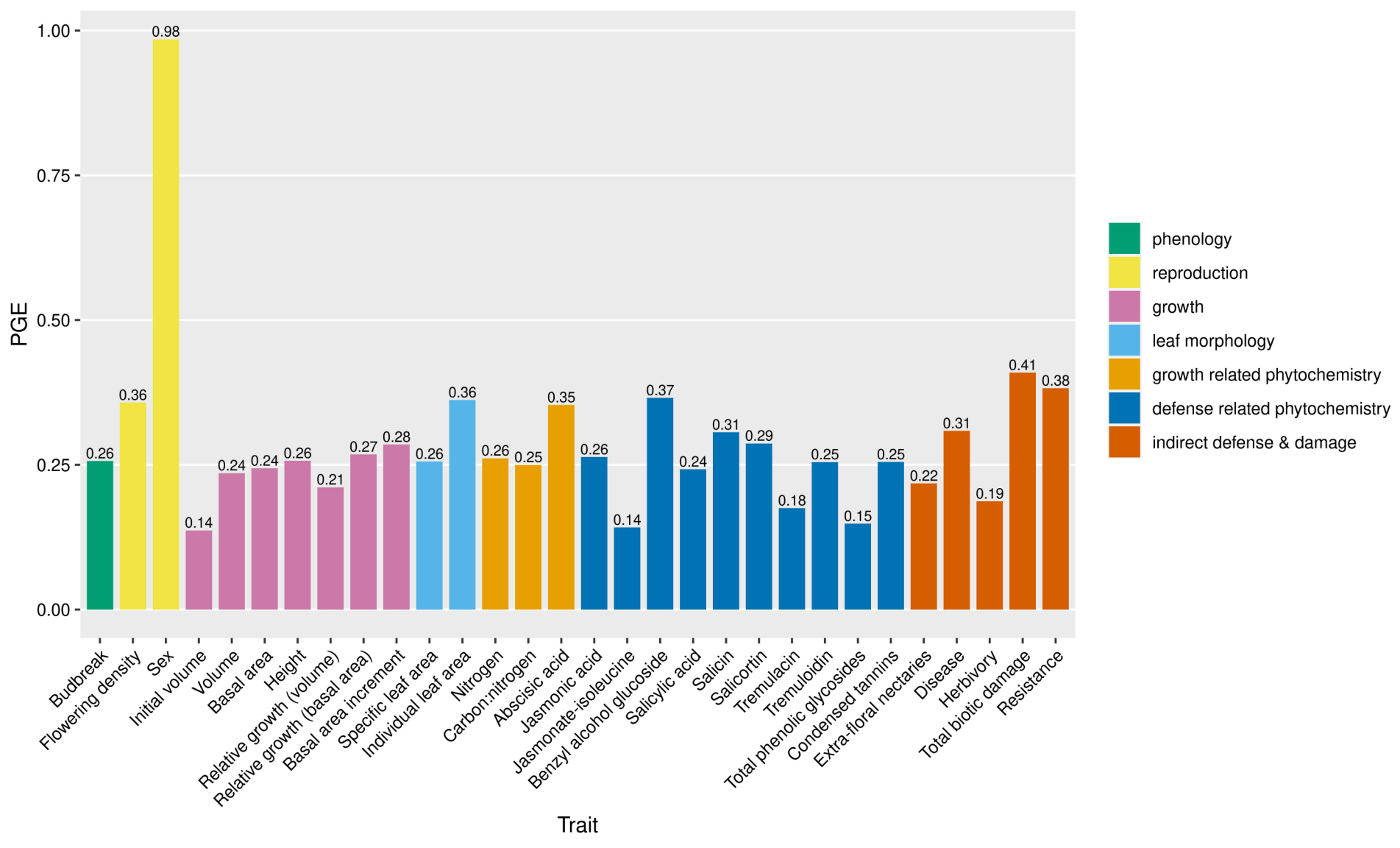

Supplement: Supplementary file 12 — Figure S4. [file ECE3-13-e10541-s013.pdf]

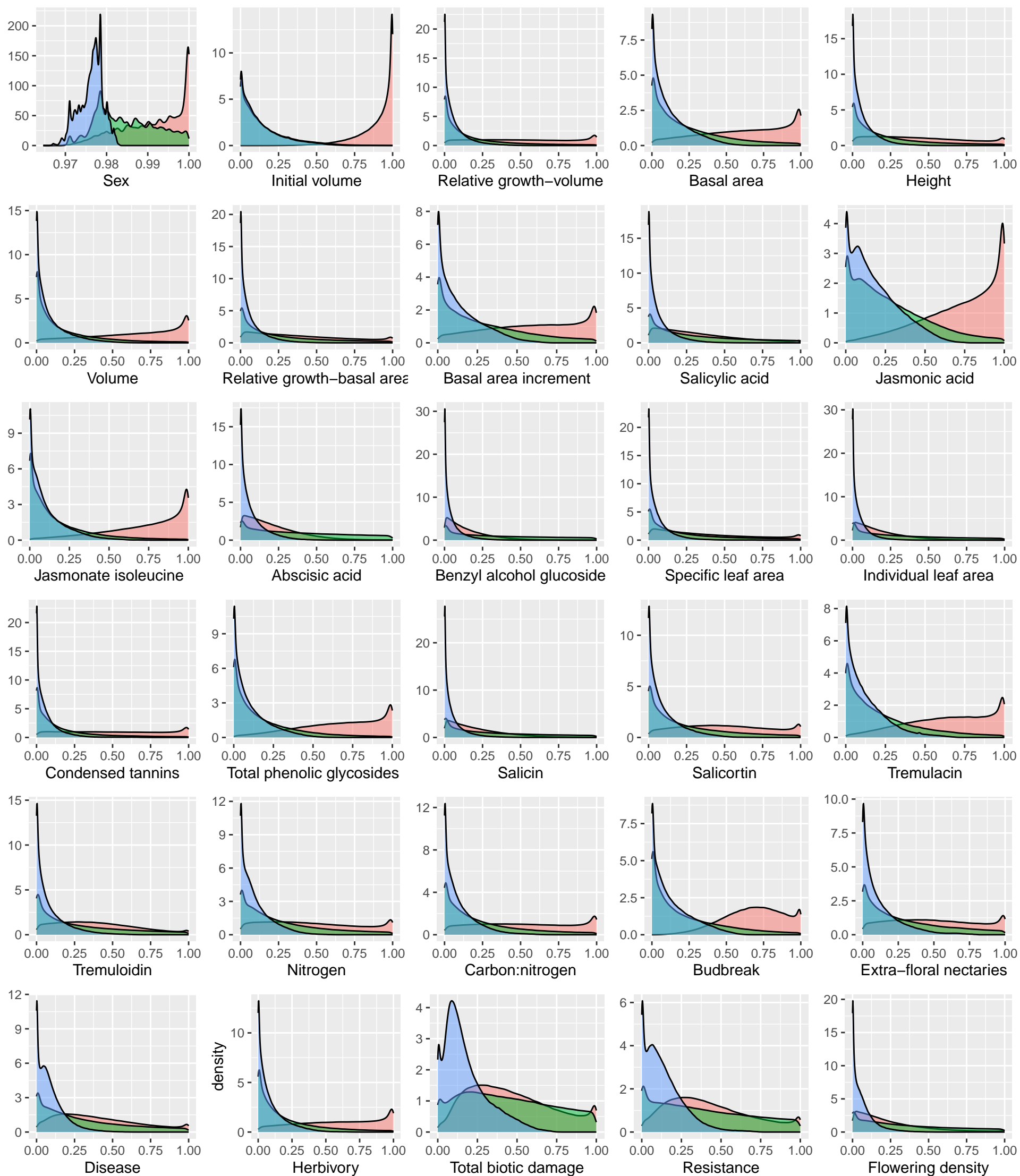

variable ■ pve ■ pge ■ nsh

Supplement: Supplementary file 13 — Figure S5. [file ECE3-13-e10541-s003.pdf]
