# Supplementary material for: Genomic and transcriptomic analyses reveal polygenic architecture for ecologically important traits in aspen (Populus tremuloides Michx.)
Source: Ecol Evol. 2023 Sep 28;13(10):e10541. doi: 10.1002/ece3.10541 (PMC10534199; doi:10.1002/ece3.10541)
Supplement: Supplementary file 14 — Tables S1.–S2. [file ECE3-13-e10541-s012.docx]

**Appendix: Supplemental Methods and Results**

**Supplemental Files List**

**Best Linear Unbiased Prediction Model Details**

Table S1 provides a detailed overview of how the best linear unbiased prediction (BLUPs) of the genetic effects for each trait to be used in the genome-wide association (GWA) analyses were obtained. Table S2 provided variance components for each model exhibiting the significant effect genet has on each trait.

Table S1 also connects the trait abbreviations used in all the GWA input (available on Dryad, DOI: <https://doi.org/10.5061/dryad.9zw3r22jr>) and results files to the trait name in the manuscript as specified in the footnote associated with the first two columns. See footnotes for detailed explanation of each column.

Table S2 provides details on the BLUP model variance components of the random effects, displaying the importance of genet effects on trait variation.

**Table S1 Tree trait abbreviations and model characteristics**

| Trait abbreviation† | Trait name† | Transformation‡ | Scaled | Linear mixed-effects model used to extract BLUPs§¶ |
| --- | --- | --- | --- | --- |
| Traits measured or calculated at a single time point | | | | |
| Vol.2012 | Initial volume | sqrt | Yes | Vol.2012~Block + Border.Tree +  Age +  (1\|Genet.SSRrev) |
| GrowBALn.1518 | Relative growth (basal area) between 2015 and 2018 | sqrt | Yes | GrowBALn.1518~Block + Border.Tree +  Age +  BA.2012sqrt + (1\|Genet.SSRrev) |
| GrowBAI.1518 | Basal area increment between 2015 and 2018 | sqrt | Yes | GrowBAI.1518~Block + Border.Tree +  Age +  BA.2012sqrt + (1\|Genet.SSRrev) |
| GrowVolLn.1516 | Relative growth (volume) between 2015 and 2016 | sqrt | Yes | GrowVolLn.1516~Block + Border.Tree +  Age +  BA.2012sqrt + (1\|Genet.SSRrev) |
| GrowVolLn.1617 | Relative growth (volume) between 2016 and 2017 | sqrt | Yes | GrowVolLn.1617~Block + Border.Tree +  Age +  BA.2012sqrt + (1\|Genet.SSRrev) |
| GrowVolLn.1718 | Relative growth (volume) between 2017 and 2018 | sqrt | Yes | GrowVolLn.1718~Block + Border.Tree +  Age +  BA.2012sqrt + (1\|Genet.SSRrev) |
| GrowVolLn.1518 | Relative growth (volume) between 2015 and 2018 | sqrt | Yes | GrowVolLn.1518~Block + Border.Tree +  Age +  BA.2012sqrt + (1\|Genet.SSRrev) |
| SA.J2017 | Salicylic acid | BC | Yes | SA.J2017~Block + Border.Tree +  Age +  BA.2012sqrt + (1\|Genet.SSRrev) |
| JA.J2017 | Jasmonic acid | BC | Yes | JA.J2017~Block + Border.Tree +  Age +  BA.2012sqrt + (1\|Genet.SSRrev) |
| JAIle.J2017 | Jasmonate-isoleucine | BC | Yes | JAIle.J2017~Block + Border.Tree +  Age +  BA.2012sqrt + (1\|Genet.SSRrev) |
| ABA.J2017 | Abscisic acid | BC | Yes | ABA.J2017~Block + Border.Tree +  Age +  BA.2012sqrt + (1\|Genet.SSRrev) |
| BAgluc.J2017 | Benzyl alcohol glucoside | BC | Yes | BAgluc.J2017~Block + Border.Tree +  Age +  BA.2012sqrt + (1\|Genet.SSRrev) |
| Traits measured at multiple time points (e.g., 2014, 2015, 2016, 2017) | | | | |
| BAsqrt | Basal area | sqrt | Yes | BAsqrt~Block + Border.Tree +  Age + BA.2012sqrt + (1\|Survey.Year) + (1\|Genet.SSRrev) |
| Height | Height | NT | Yes | Height~Block + Border.Tree +  Age + BA.2012sqrt + (1\|Survey.Year) + (1\|Genet.SSRrev) |
| Vol | Volume | sqrt | Yes | Vol~Block + Border.Tree +  Age + BA.2012sqrt +  (1\|Survey.Year) + (1\|Genet.SSRrev) |
| GrowBALn | Relative growth (basal area) | sqrt | Yes | GrowBALn~Block + Border.Tree +  Age +  BA.2012sqrt + (1\|Survey.Year) + (1\|Genet.SSRrev) |
| GrowBAI | Basal area increment | sqrt | Yes | GrowBAI~Block + Border.Tree +  Age +  BA.2012sqrt + (1\|Survey.Year) + (1\|Genet.SSRrev) |
| SLA | Specific leaf area | NT | Yes | SLA~Block + Border.Tree +  Age + BA.2012sqrt +  (1\|Survey.Year) + (1\|Genet.SSRrev) |
| ALA | Average individual leaf area | NT | Yes | ALA~Block + Border.Tree +  Age + BA.2012sqrt +  (1\|Survey.Year) + (1\|Genet.SSRrev) |
| CT | Condensed tannins | NT | Yes | CT~Block + Border.Tree +  Age + BA.2012sqrt +  (1\|Survey.Year) + (1\|Genet.SSRrev) |
| PG | Total phenolic glycosides | BC | Yes | PG~Block + Border.Tree +  Age + BA.2012sqrt +  (1\|Survey.Year) + (1\|Genet.SSRrev) |
| Scin | Salicin | BC | Yes | Scin~Block + Border.Tree +  Age + BA.2012sqrt +  (1\|Survey.Year) + (1\|Genet.SSRrev) |
| Scort | Salicortin | BC | Yes | Scort~Block + Border.Tree +  Age + BA.2012sqrt +  (1\|Survey.Year) + (1\|Genet.SSRrev) |
| Tcin | Tremulacin | BC | Yes | Tcin~Block + Border.Tree +  Age + BA.2012sqrt +  (1\|Survey.Year) + (1\|Genet.SSRrev) |
| Tdin | Tremuloidin | BC | Yes | Tdin~Block + Border.Tree +  Age + BA.2012sqrt +  (1\|Survey.Year) + (1\|Genet.SSRrev) |
| Npct | Nitrogen | NT | Yes | Npct~Block + Border.Tree +  Age + BA.2012sqrt +  (1\|Survey.Year) + (1\|Genet.SSRrev) |
| CN | Carbon:nitrogen | NT | Yes | CN~Block + Border.Tree +  Age + BA.2012sqrt +  (1\|Survey.Year) + (1\|Genet.SSRrev) |
| BBreakDegDayRT | Budbreak | RT | No | BBreakDegDayRT~Block + Border.Tree +  Age +  BA.2012sqrt + (1\|Survey.Year) + (1\|Genet.SSRrev) |
| EFNMean | Extra-floral nectaries | NT | Yes | EFNMean~Block + Border.Tree +  Age +  BA.2012sqrt + (1\|Survey.Year) + (1\|Genet.SSRrev) |
| DiseaseEdgePct | Disease | BC | Yes | DiseaseEdgePct~Block + Border.Tree +  Age +  BA.2012sqrt + (1\|Survey.Year) + (1\|Genet.SSRrev) |
| ScrapHolePct | Herbivory | BC | Yes | ScrapHolePct~Block + Border.Tree +  Age +  BA.2012sqrt + (1\|Survey.Year) + (1\|Genet.SSRrev) |
| DamagePct | Total biotic damage | BC | Yes | DamagePct~Block + Border.Tree +  Age +  BA.2012sqrt + (1\|Survey.Year) + (1\|Genet.SSRrev) |
| ResistancePct | Resistance | NT | Yes | ResistancePct~Block + Border.Tree +  Age +  BA.2012sqrt + (1\|Survey.Year) + (1\|Genet.SSRrev) |
| Flprev | Flowering density | NT | No | Flprev>0~Block + Border.Tree +  Age + BA.2012sqrt + (1\|Survey.Year) + (1\|Genet.SSRrev) |

† Trait abbreviation corresponds to the trait labels in input files for genome-wide association analyses and trait name corresponds to the labels used in the article’s tables and figures.

‡ transformation: NT= not transformed, BC= BoxCox transformation (MASS package for R,(Venables, W. N., & Ripley, 2002), RT= rank transformed, sqrt= square root transformed.

§ The format follows the lme4 package notation for writing model parameter commands; parentheses indicate a random variable.

¶ Broad-sense heritabilities are calculated by dividing the genet residual variance by the total residual variance. Since all environmental variables (e.g., initial tree size, etc.) should be counted as part of the total residual variance, the model was adjusted to make all environmental variables random instead of fixed. The residual variance estimates extracted from these adjusted models were used to calculate broad-sense heritabilities for all traits.

**Table S2**  BLUP model variance components of the random effects

| **Trait name** | **Trait abbreviation** | **Random effect** | **Variance** | **Standard deviation** |
| --- | --- | --- | --- | --- |
| **Traits measured or calculated at a single time point** | | | | |
| Vol.2012 | Initial volume | Genet | 0.56 | 0.75 |
|  |  | Residual | 0.37 | 0.61 |
| GrowBALn.1518 | Relative growth (basal area) between 2015 and 2018 | Genet | 0.25 | 0.50 |
|  |  | Residual | 0.28 | 0.53 |
| GrowBAI.1518 | Basal area increment between 2015 and 2018 | Genet | 0.39 | 0.62 |
|  |  | Residual | 0.18 | 0.42 |
| GrowVolLn.1516 | Relative growth (volume) between 2015 and 2016 | Genet | 0.25 | 0.51 |
|  |  | Residual | 0.31 | 0.56 |
| GrowVolLn.1617 | Relative growth (volume) between 2016 and 2017 | Genet | 0.30 | 0.54 |
|  |  | Residual | 0.37 | 0.61 |
| GrowVolLn.1718 | Relative growth (volume) between 2017 and 2018 | Genet | 0.27 | 0.52 |
|  |  | Residual | 0.49 | 0.70 |
| GrowVolLn.1518 | Relative growth (volume) between 2015 and 2018 | Genet | 0.23 | 0.48 |
|  |  | Residual | 0.26 | 0.51 |
| SA.J2017 | Salicylic acid | Genet | 0.57 | 0.75 |
|  |  | Residual | 0.41 | 0.64 |
| JA.J2017 | Jasmonic acid | Genet | 0.62 | 0.78 |
|  |  | Residual | 0.38 | 0.62 |
| JAIle.J2017 | Jasmonate-isoleucine | Genet | 0.54 | 0.73 |
|  |  | Residual | 0.42 | 0.65 |
| ABA.J2017 | Abscisic acid | Genet | 0.51 | 0.71 |
|  |  | Residual | 0.52 | 0.72 |
| BAgluc.J2017 | Benzyl alcohol glucoside | Genet | 0.79 | 0.89 |
|  |  | Residual | 0.24 | 0.49 |
| **Traits measured at multiple time points (e.g., 2014, 2015, 2016, 2017)** | | | | |
| BAsqrt | Basal area | Genet | 0.14 | 0.37 |
|  |  | Survey Year | 0.39 | 0.63 |
|  |  | Residual | 0.11 | 0.33 |
| Height | Height | Genet | 0.17 | 0.41 |
|  |  | Survey Year | 0.43 | 0.66 |
|  |  | Residual | 0.19 | 0.43 |
| Vol | Volume | Genet | 0.13 | 0.36 |
|  |  | Survey Year | 0.46 | 0.68 |
|  |  | Residual | 0.12 | 0.34 |
| GrowBALn | Relative growth (basal area) | Genet | 0.07 | 0.27 |
|  |  | Survey Year | 0.36 | 0.60 |
|  |  | Residual | 0.28 | 0.53 |
| GrowBAI | Basal area increment | Genet | 0.26 | 0.51 |
|  |  | Survey Year | 0.04 | 0.20 |
|  |  | Residual | 0.24 | 0.49 |
| SLA | Specific leaf area | Genet | 0.24 | 0.49 |
|  |  | Survey Year | 0.16 | 0.40 |
|  |  | Residual | 0.41 | 0.64 |
| ALA | Average individual leaf area | Genet | 0.55 | 0.74 |
|  |  | Survey Year | 0.068 | 0.26 |
|  |  | Residual | 0.33 | 0.58 |
| CT | Condensed tannins | Genet | 0.35 | 0.59 |
|  |  | Survey Year | 0.38 | 0.61 |
|  |  | Residual | 0.34 | 0.58 |
| PG | Total phenolic glycosides | Genet | 0.58 | 0.76 |
|  |  | Survey Year | 0.24 | 0.49 |
|  |  | Residual | 0.23 | 0.48 |
| Scin | Salicin | Genet | 0.39 | 0.62 |
|  |  | Survey Year | 0.08 | 0.28 |
|  |  | Residual | 0.53 | 0.73 |
| Scort | Salicortin | Genet | 0.50 | 0.70 |
|  |  | Survey Year | 0.32 | 0.56 |
|  |  | Residual | 0.22 | 0.47 |
| Tcin | Tremulacin | Genet | 0.68 | 0.82 |
|  |  | Survey Year | 0.12 | 0.35 |
|  |  | Residual | 0.22 | 0.47 |
| Tdin | Tremuloidin | Genet | 0.65 | 0.81 |
|  |  | Survey Year | 0.01 | 0.07 |
|  |  | Residual | 0.36 | 0.60 |
| Npct | Nitrogen | Genet | 0.22 | 0.47 |
|  |  | Survey Year | 0.25 | 0.50 |
|  |  | Residual | 0.45 | 0.67 |
| CN | Carbon:nitrogen | Genet | 0.23 | 0.48 |
|  |  | Survey Year | 0.29 | 0.54 |
|  |  | Residual | 0.42 | 0.65 |
| BBreakDegDayRT | Budbreak | Genet | 240,000 | 490 |
|  |  | Survey Year | 1900 | 44 |
|  |  | Residual | 44,000 | 210 |
| EFNMean | Extra-floral nectaries | Genet | 0.62 | 0.79 |
|  |  | Survey Year | 0.034 | 0.19 |
|  |  | Residual | 0.34 | 0.58 |
| DiseaseEdgePct | Disease | Genet | 0.41 | 0.64 |
|  |  | Survey Year | 0.056 | 0.24 |
|  |  | Residual | 0.51 | 0.71 |
| ScrapHolePct | Herbivory | Genet | 0.14 | 0.37 |
|  |  | Survey Year | 0.28 | 0.53 |
|  |  | Residual | 0.55 | 0.74 |
| DamagePct | Total biotic damage | Genet | 0.34 | 0.59 |
|  |  | Survey Year | 0.0028 | 0.053 |
|  |  | Residual | 0.62 | 0.79 |
| ResistancePct | Resistance | Genet | 0.44 | 0.66 |
|  |  | Survey Year | 0 | 0 |
|  |  | Residual | 0.59 | 0.77 |
| Flprev | Flowering density | Genet | 11 | 3.3 |
|  |  | Survey Year | 0.037 | 0.19 |

**Additional Methods and Results for Single-locus Genome-wide Association Analyses**
